# A CT-based multi-scale fusion model with SHAP interpretation for preoperative differentiation between lung adenocarcinoma in situ/minimally invasive adenocarcinoma and invasive adenocarcinoma: a multicenter study

**DOI:** 10.3389/fonc.2026.1824735

**Published:** 2026-05-12

**Authors:** Ning Dong, Shihang Sun, Zhongwei Li, Zhenjie Cong, Yi Lin, Jingxian Chen, Hu Zhang, Yuxin Liu, Shuxia Li, Jing Xu, Demin Kong, Qingfeng Yin

**Affiliations:** 1 Department of Radiology, Yantaishan Hospital, Yantai, China; 2 Department of Medical Imaging, Weifang Yidu Central Hospital, Qingzhou, China; 3 Department of Radiology, Affiliated Hospital of Binzhou Medical University, Binzhou, China; 4 Department of Radiology, Zibo Central Hospital, Zibo, China

**Keywords:** Deep learning, ground-glass nodules, habitat, lung adenocarcinoma, SHAP

## Abstract

**Objective:**

To develop a multi-scale model integrating radiomics, habitat, deep learning, and clinical information for preoperative precise differentiation between lung adenocarcinoma in situ/minimally invasive adenocarcinoma and invasive adenocarcinoma in ground-glass nodules (GGNs).

**Methods:**

A total of 621 patients with GGNs from two centers were retrospectively enrolled. Radiomics, habitat, deep learning (2D, 2.5D, and 3D), and clinic models were constructed separately. Early fusion (feature-level fusion) and late fusion (decision-level fusion) strategies were systematically compared. The SHapley Additive exPlanations method was applied for interpretability analysis of the optimal model.

**Results:**

The early fusion model demonstrated superior performance, achieving areas under the receiver operating characteristic curves of 0.988, 0.903, and 0.872 in the training, internal validation, and external testing cohorts, respectively. Among the deep learning architectures, the 2.5D ResNet50 achieved the best performance.

**Conclusions:**

This multi-scale early fusion model provides robust support for preoperative precise diagnosis and individualized treatment decision-making for GGNs.

## 1 Introduction

Lung cancer remains one of the most prevalent malignancies worldwide, with the highest incidence and mortality rates, posing a severe threat to human health (1). With the widespread application of computed tomography (CT) in lung cancer screening, the detection rate of ground-glass nodules (GGNs) has significantly increased (2). Among these, GGNs associated with lung adenocarcinoma can be classified according to their degree of invasion into adenocarcinoma *in situ* (AIS), minimally invasive adenocarcinoma (MIA), and invasive adenocarcinoma (IAC) (3). AIS/MIA have a fairly inert nature in their biology, and they have a good prognosis with almost all patients surviving five years after diagnosis (4); sublobar resection (e.g., wedge resection or segmentectomy) has been reported to cure such lesions (5). Conversely, IAC shows a 5-year disease-free survival rate ranging from 38% to 86% (6) and requires a conventional lobectomy with systematic removal of lymph nodes (7). Hence, correct preoperative distinction between AIS/MIA and IAC is significantly important to prevent undertreatment or overtreatment, improve the prognosis of the patient, and maximize the use of healthcare resources.

Radiomics is an emerging approach that has gained popularity in recent years, translating visually imperceptible imaging features into mineable quantitative data and offering novel diagnostic tools (8–13). Despite the promising application opportunities of radiomics, conventional radiomics techniques consider tumors as uniform objects, disregarding intratumoral spatial heterogeneity (14–16). The habitat analysis techniques have the ability to divide the habitat into subregions that have the same imaging properties and therefore provide a precise description of the spatial heterogeneity of the tumor (17–19). At the same time, deep learning (DL) has proven to be more effective in the prediction of pathological subtypes of GGNs (20, 21). To our knowledge, the simultaneous integration of radiomics, habitat, DL, and clinical information for distinguishing AIS/MIA from IAC remains unexplored in the existing literature. Given this background, this study aimed to construct a multi-scale fusion model integrating radiomics, habitat, DL, and clinical information for precise preoperative differentiation between AIS/MIA and IAC. Through a multicenter, large-sample retrospective cohort design, this study provides a comprehensive, objective, and reliable solution for precise preoperative diagnosis of GGNs, facilitating individualized clinical treatment decision-making.

## 2 Materials and methods

**Figure 1:**
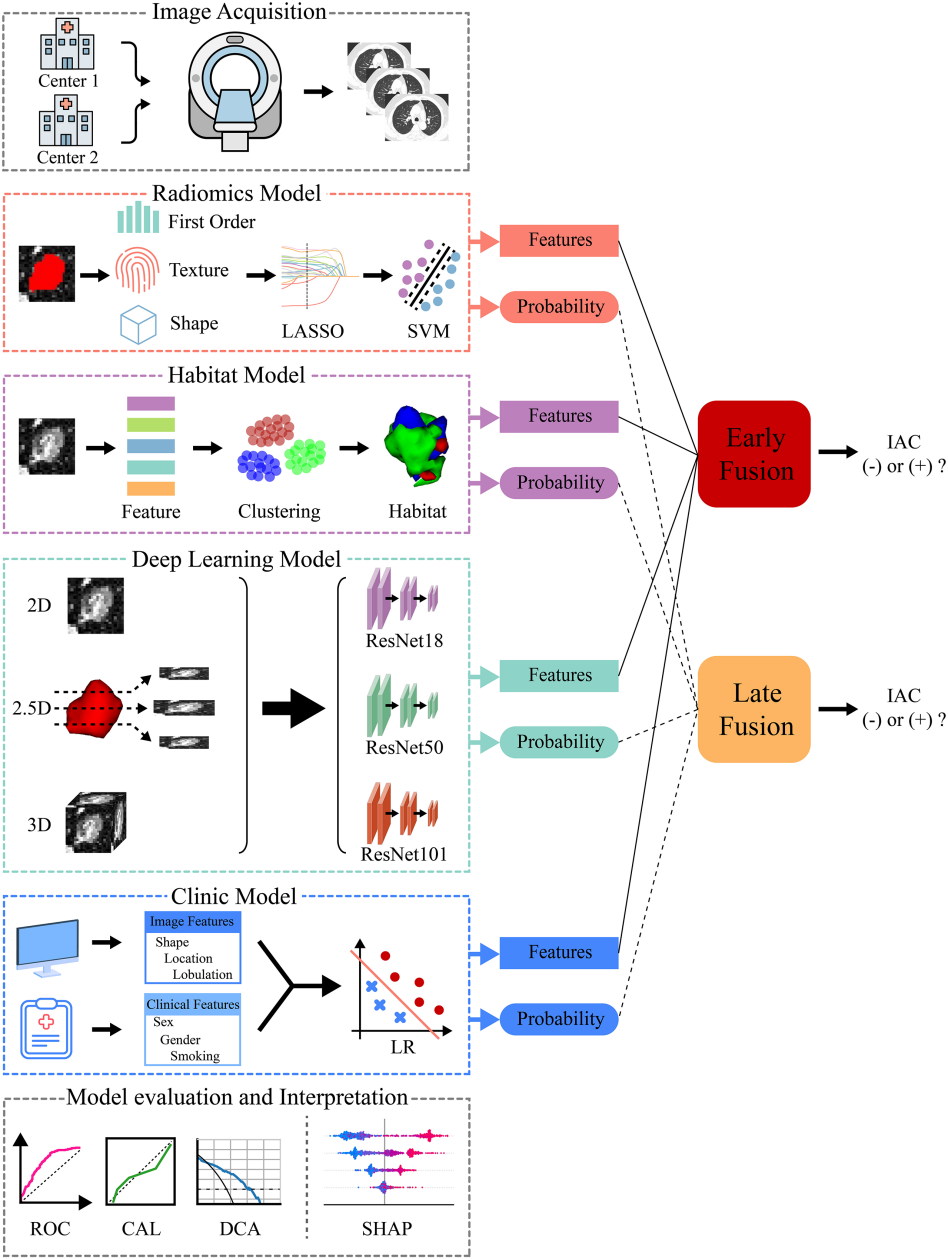
Overall workflow of this study. In this study, patient CT data were first acquired from two medical centers, followed by the independent development of radiomics, habitat, deep learning, and clinic models. The features and probabilities generated by each model were integrated using early and late fusion strategies, respectively, to differentiate invasive adenocarcinoma lesions. The predictive performance and clinical utility of the models were comprehensively evaluated via receiver operating characteristic curves, calibration curves, and decision curve analysis. The SHapley Additive exPlanations method was also used to interpret the fusion model. This clarified the contribution of individual features to the model’s decisions. CAL, calibration; DCA, decision curve analysis; IAC, invasive adenocarcinoma; LASSO, least absolute shrinkage and selection operator; LR, logistic regression; ROC, receiver operating characteristic; SHAP, SHapley Additive exPlanations; SVM, support vector machine.

This study has been approved by the ethics committees of Yantaishan Hospital (LL-2026-024-L) and the Affiliated Hospital of Binzhou Medical University (2025-KT-62). Given its retrospective design, the requirement for informed consent has been waived. The entire research process strictly adhered to the ethical principles of the Declaration of Helsinki. **Figure 1** presents the overall workflow of this study.

### 2.1 Patients

Clinical data were retrospectively collected from patients who underwent surgical resection for GGNs between June 2020 and July 2023 at Yantaishan Hospital (Center 1) and the Affiliated Hospital of Binzhou Medical University (Center 2).

**Figure 2:**
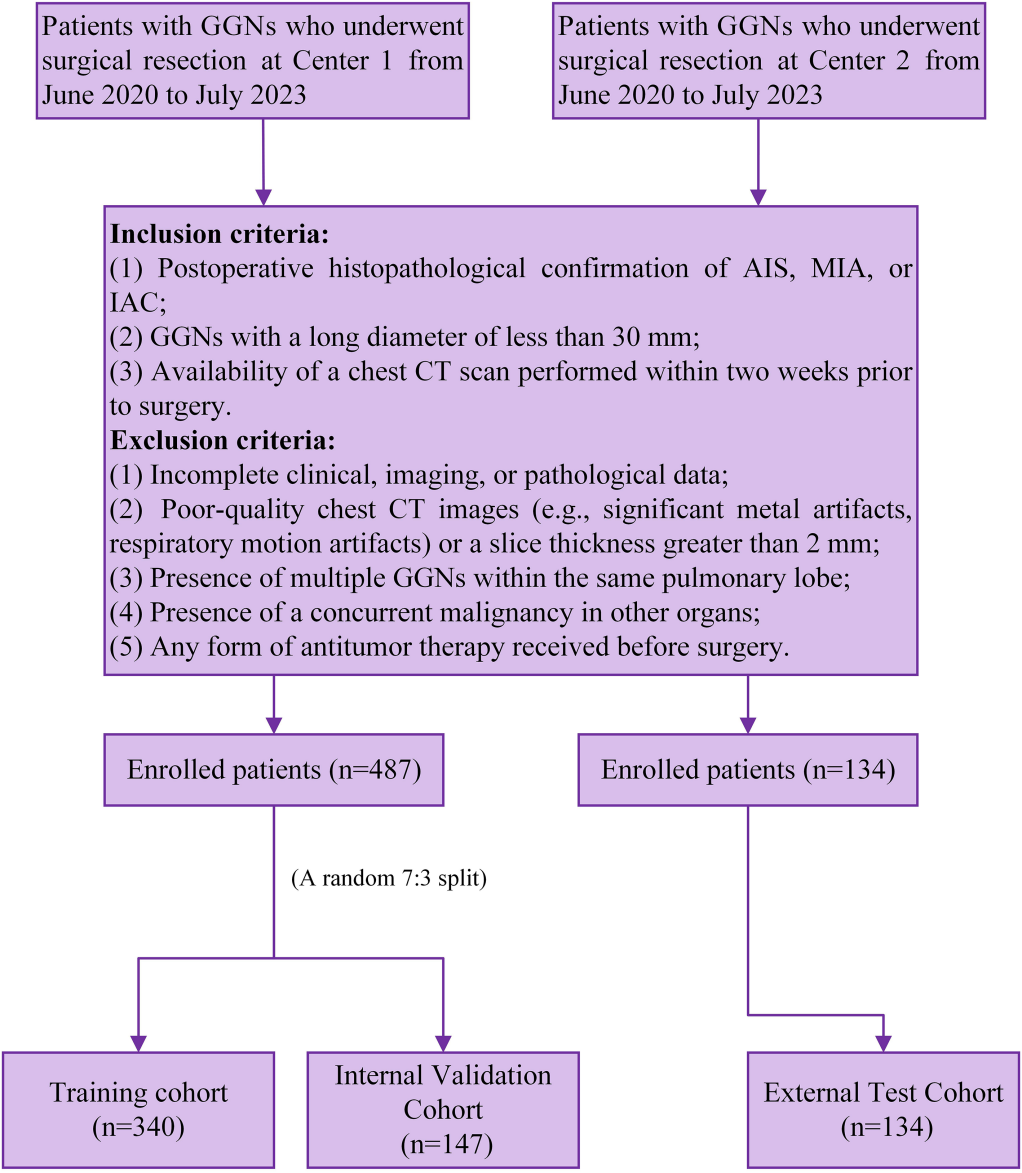
Flowchart of case screening for this study. AIS, adenocarcinoma *in situ*; CT, computed tomography; GGNs, ground-glass nodules; IAC, invasive adenocarcinoma; MIA, minimally invasive adenocarcinoma.

Following the strenuous screening (the inclusion and exclusion criteria are presented in **Supplementary S1**), the final analysis involved 621 GGNs of 621 patients. The patients at Center 1 (n=487) were separated into training (n=340) and internal validation (n=147) cohorts at a ratio of 7:3, whereas the patients at Center 2 (n=134) served as the external test cohort. The entire patient selection flowchart is illustrated in **Figure 2**.

### 2.2 Image acquisition and preprocessing

All chest CT examinations were non-contrast scans performed with patients in the supine position during end-inspiratory breath-holding, with scanning range from the lung apex to the costophrenic angle. The specific scanners and parameters are provided in **Supplementary Table 1**.

To enhance reproducibility, reliability, and clinical applicability, CT images were first standardized using a window width of 1200 Hounsfield Units (HU) and a window level of −600 HU, followed by resampling to a voxel size of 1 mm × 1 mm × 1 mm using bicubic spline interpolation.

### 2.3 ROI segmentation

CT images were imported into ITK-SNAP software (version 3.8.0; http://www.itksnap.org). A radiologist A with 5 years of chest CT experience delineated the region of interest (ROI) layer-by-layer while blinded to pathological results, avoiding major vessels and bronchi. Subsequently, radiologist B with 10 years of chest CT experience reviewed the delineations. In case of disagreement, consensus was reached through joint review.

### 2.4 Habitat generation

To generate tumor habitats, 19 local features were extracted from each voxel within the GGNs (a visual example of local features is shown in **Supplementary Figure 1**). Subsequently, K-means clustering was applied to identify habitat subregions with similar imaging attributes. The number of clusters was traversed from 2 to 9, with the optimal cluster number determined by maximizing the Calinski-Harabasz index. A detailed habitat generation workflow is provided in **Supplementary S2**.

### 2.5 Extraction and selection of radiomics and habitat features

This study strictly followed the Imaging Biomarker Standardization Initiative (IBSI) guidelines (8). Radiomics features were extracted from tumor ROIs and habitat subregions using PyRadiomics (version 3.0.1). To ensure feature stability, only those with both intraclass and interclass correlation coefficients > 0.75 were retained. To eliminate batch effects arising from differences in data acquisition equipment and scanning parameters across two centers, ComBat harmonization was applied to the radiomics features to achieve cross-center data calibration (22). All features were then Z-score standardized using the mean and standard deviation of the training cohort to eliminate dimensional differences. Feature selection proceeded in several steps: (1) removal of redundant variables with Pearson correlation ≥ 0.9; (2) selection of the top 30 features by the minimum Redundancy Maximum Relevance algorithm; (3) final dimensionality reduction via the least absolute shrinkage and selection operator (LASSO) regression with 10-fold cross-validation. Further details are provided in **Supplementary S3**.

### 2.6 Deep learning model training and feature extraction

To systematically evaluate the performance of different dimensions and network architectures, this study constructed a multi-dimensional, multi-architecture DL model system. Specifically, we systematically trained DL models based on three classic convolutional neural network architectures—ResNet18, ResNet50, and ResNet101—under 2D, 2.5D, and 3D image input modes (detailed training process in **Supplementary S4**).

After model training, DL features were extracted from the penultimate average pooling layer, and dimensionality reduction and feature selection were completed following the workflow described in “Section 2.5”. Finally, a support vector machine (SVM) was employed to construct prediction models. By comparing the area under the curve (AUC) across different DL models on the internal validation cohort, we identified the optimal model architecture.

### 2.7 Constructions of clinic, radiomics, habitat, and fusion models

In constructing the Clinic model, we first performed univariate logistic regression on all clinical and imaging variables to screen potential risk factors with p < 0.05. Multivariate logistic regression was then used to identify independent risk factors for IAC. Finally, an SVM algorithm was employed to build the Clinic model.

For the Radiomics and Habitat models, we constructed prediction models using SVM based on the optimal feature subsets selected by LASSO.

In fusion model construction, this study systematically compared early fusion (feature-level fusion) and late fusion (decision-level fusion) strategies. In early fusion, clinical, radiomics, habitat, and optimal DL features were concatenated to form a high-dimensional joint feature space. Subsequently, dimensionality reduction and feature selection were performed following the pipeline described in “Section 2.5”, after which an SVM was trained for the early fusion (Early) model. In late fusion, a stacking ensemble learning framework was adopted: prediction probabilities from Clinic, Radiomics, Habitat, and optimal DL models served as meta-features input to a second-layer SVM classifier, achieving decision-level information fusion and constructing the late fusion (Late) model. To ensure methodological rigor and prevent information leakage, these meta-features were generated using 5-fold out-of-fold (OOF) predictions. Specifically, the training set was partitioned into five folds; in each iteration, each base model was trained on four folds and used to predict class probabilities for the remaining held-out fold. After all five iterations, the concatenated OOF probabilities were used to train the SVM meta-learner. During test-time inference, all base models were retrained on the entire training set, and their predicted probabilities on the independent test set were then fed into the trained SVM meta-learner.

### 2.8 Model performance evaluation and interpretation

Model discrimination performance was evaluated using the receiver operating characteristic (ROC) curve-related metrics, with the DeLong test comparing AUC differences between models. Model calibration was assessed through calibration curves combined with the Hosmer-Lemeshow goodness-of-fit test to quantify the agreement between predicted and observed probabilities. Decision curve analysis (DCA) evaluated clinical net benefit across different probability thresholds.

Additionally, we employed SHAP to interpret predictions of the fusion model. By quantifying each feature’s contribution to specific predictions, SHAP reveals the “black box” decision-making process of the model. The algorithm type, feature sampling strategy, and calculation parameters of the SHAP analysis are detailed in **Supplementary S5**.

### 2.9 Statistical analysis

For continuous variables, the Shapiro-Wilk test assessed normality; normally distributed variables were expressed as mean ± standard deviation, with an independent samples t-test or one-way ANOVA for group comparisons. Categorical variables were described as frequency (percentage), with the χ² test or Fisher’s exact test for group comparisons. All statistical tests were two-tailed, with p < 0.05 considered statistically significant. Statistical analyses were performed using SPSS 26.0 and Python 3.9.7.

## 3 Results

### 3.1 Patients

This study included 621 GGNs from 621 patients from two medical centers. **Supplementary Table 2** summarizes baseline characteristics across the three cohorts, showing no significant statistical differences, indicating good comparability of cohort division. **Table 1** systematically compared clinical and imaging features between AIS/MIA and IAC patients across different cohorts. Results demonstrated that lobulation, spiculation, margin, vessel changes, pleural retraction, shape, long diameter, short diameter, age, and CT value showed statistically significant inter-group differences across all three cohorts.

**Table 1:** Features of patients with AIS/MIA versus IAC in different cohorts.

Features	Training cohort (n = 340)		P	Internal validation cohort (n = 147)		P	External test cohort (n = 134)		P
	AIS/MIA (n = 185)	IAC (n = 155)		AIS/MIA (n = 78)	IAC (n = 69)		AIS/MIA (n = 81)	IAC (n = 53)	
Gender [n (%)]			0.981			0.938			0.971
Male	61 (32.97)	50 (32.26)		23 (29.49)	19 (27.54)		26 (32.10)	18 (33.96)	
Female	124 (67.03)	105 (67.74)		55 (70.51)	50 (72.46)		55 (67.90)	35 (66.04)	
Smoking [n (%)]			0.202			0.309			0.217
No	166 (89.73)	131 (84.52)		70 (89.74)	57 (82.61)		73 (90.12)	43 (81.13)	
Yes	19 (10.27)	24 (15.48)		8 (10.26)	12 (17.39)		8 (9.88)	10 (18.87)	
Location [n (%)]			0.753			0.447			0.984
RUL	57 (30.81)	49 (31.61)		26 (33.33)	21 (30.43)		28 (34.57)	16 (30.19)	
RML	11 (5.95)	9 (5.81)		3 (3.85)	7 (10.14)		6 (7.41)	4 (7.55)	
RLL	27 (14.59)	30 (19.35)		14 (17.95)	15 (21.74)		14 (17.28)	9 (16.98)	
LUL	57 (30.81)	40 (25.81)		24 (30.77)	15 (21.74)		24 (29.63)	18 (33.96)	
LLL	33 (17.84)	27 (17.42)		11 (14.10)	11 (15.94)		9 (11.11)	6 (11.32)	
Lobulation [n (%)]			<0.001			<0.001			<0.001
No	72 (38.92)	5 (3.23)		32 (41.03)	7 (10.14)		32 (39.51)	3 (5.66)	
Yes	113 (61.08)	150 (96.77)		46 (58.97)	62 (89.86)		49 (60.49)	50 (94.34)	
Spiculation [n (%)]			<0.001			<0.001			<0.001
No	148 (80.00)	70 (45.16)		56 (71.79)	29 (42.03)		67 (82.72)	28 (52.83)	
Yes	37 (20.00)	85 (54.84)		22 (28.21)	40 (57.97)		14 (17.28)	25 (47.17)	
Margin [n (%)]			<0.001			0.034			0.037
Clear	134 (72.43)	83 (53.55)		54 (69.23)	35 (50.72)		57 (70.37)	27 (50.94)	
Unclear	51 (27.57)	72 (46.45)		24 (30.77)	34 (49.28)		24 (29.63)	26 (49.06)	
Vessel changes [n (%)]			<0.001			<0.001			<0.001
No	99 (53.51)	16 (10.32)		39 (50.00)	11 (15.94)		41 (50.62)	7 (13.21)	
Yes	86 (46.49)	139 (89.68)		39 (50.00)	58 (84.06)		40 (49.38)	46 (86.79)	
Bubble lucency [n (%)]			0.017			0.003			0.461
No	139 (75.14)	97 (62.58)		63 (80.77)	39 (56.52)		61 (75.31)	36 (67.92)	
Yes	46 (24.86)	58 (37.42)		15 (19.23)	30 (43.48)		20 (24.69)	17 (32.08)	
Pleural retraction [n (%)]			<0.001			0.002			<0.001
No	142 (76.76)	68 (43.87)		53 (67.95)	28 (40.58)		66 (81.48)	20 (37.74)	
Yes	43 (23.24)	87 (56.13)		25 (32.05)	41 (59.42)		15 (18.52)	33 (62.26)	
Shape [n (%)]			<0.001			<0.001			<0.001
Round	115 (62.16)	27 (17.42)		46 (58.97)	18 (26.09)		41 (50.62)	6 (11.32)	
Irregular	70 (37.84)	128 (82.58)		32 (41.03)	51 (73.91)		40 (49.38)	47 (88.68)	
Long diameter (mm)	9.25 ± 3.63	15.66 ± 5.46	<0.001	9.22 ± 2.88	14.47 ± 5.67	<0.001	9.04 ± 3.11	16.32 ± 6.39	<0.001
Short diameter (mm)	7.43 ± 2.45	11.15 ± 4.22	<0.001	7.07 ± 2.03	10.92 ± 4.32	<0.001	6.64 ± 1.99	10.86 ± 3.80	<0.001
Age (years)	51.03 ± 12.24	60.35 ± 8.85	<0.001	53.97 ± 10.77	61.13 ± 9.90	<0.001	53.38 ± 11.45	59.38 ± 9.32	0.002
NSE (ng/ml)	14.53 ± 5.31	15.74 ± 4.81	0.007	14.44 ± 4.39	15.73 ± 6.58	0.413	15.22 ± 4.83	14.38 ± 3.58	0.446
CEA (ng/ml)	1.77 ± 1.17	2.11 ± 1.31	0.002	1.90 ± 1.41	2.05 ± 1.22	0.154	1.65 ± 1.11	3.67 ± 12.99	0.042
CT value (HU)	-486.53 ± 180.75	-338.35 ± 184.68	<0.001	-468.64 ± 168.99	-325.38 ± 203.76	<0.001	-470.88 ± 173.67	-321.30 ± 166.69	<0.001

AIS, adenocarcinoma *in situ*; CEA, carcinoembryonic antigen; CT, computed tomography; HU, Hounsfield units; IAC, invasive adenocarcinoma; LLL, left lower lobe; LUL, left upper lobe; MIA, minimally invasive adenocarcinoma; NSE, neuron-specific enolase; RLL, right lower lobe; RML, right middle lobe; RUL, right upper lobe.

### 3.2 Habitat generation

To determine the optimal cluster number, this study traversed the range of 2–9 using the Calinski-Harabasz score as the evaluation criterion. **Supplementary Figure 2** shows that this index peaked at 3 clusters, establishing the optimal cluster number as 3. Accordingly, habitat subregions were designated as habitat 1 (h1), habitat 2 (h2), and habitat 3 (h3).

### 3.3 Feature selection and model construction

A total of 1,834 radiomics features were extracted from the tumor ROI and each habitat subregion. After LASSO feature selection, 18 radiomics features were retained for the Radiomics model construction (**Supplementary Figure 3**), and 15 habitat features for the Habitat model construction (**Supplementary Figure 4**).

By comparing AUCs of DL models on the internal validation cohort, 2.5D ResNet50 achieved the highest value and was established as the optimal DL model (**Supplementary Table 3**; **Supplementary Figure 5**). The training and validation loss curves of the optimal DL model are presented in **Supplementary Figure 6**. A total of 2,048 DL features were extracted from its penultimate average pooling layer for subsequent analysis.

In the Early model construction, LASSO selection of concatenated high-dimensional features ultimately identified 19 non-zero coefficient features for the prediction model (**Supplementary Figure 7**).

Multivariate logistic regression identified two independent risk factors (long diameter and CT value) for the construction of the Clinic model (**Table 2**).

**Table 2:** Univariate and multivariate logistic regression analysis of clinical and imaging variables.

Features	Univariate logistic regression		Multivariate logistic regression	
	OR (95% CI)	P	OR (95% CI)	P
Gender	0.847 (0.681–1.053)	0.210		
Location	0.934 (0.868–1.004)	0.120		
Bubble lucency	1.261 (0.911–1.745)	0.240		
Smoking	1.263 (0.763–2.094)	0.447		
Lobulation	1.327 (1.081–1.629)	0.023	0.918 (0.480–1.754)	0.828
Margin	1.412 (1.045–1.908)	0.060		
Vessel changes	1.616 (1.289–2.026)	0	1.815 (1.060–3.108)	0.069
Shape	1.829 (1.432–2.335)	0	1.782 (1.036–3.068)	0.080
Pleural retraction	2.023 (1.489–2.748)	0	0.983 (0.584–1.655)	0.958
Spiculation	2.297 (1.662–3.177)	0	0.753 (0.437–1.297)	0.391
Long diameter	1.024 (1.010–1.039)	0.004	1.220 (1.142–1.303)	0
Short diameter	1.022 (1.003–1.041)	0.054		
Age	1.000 (0.997–1.003)	0.934		
NSE	0.994 (0.983–1.005)	0.398		
CEA	1.000 (0.925–1.081)	0.994		
CT value	1.001 (1.001–1.001)	0	1.007 (1.006–1.008)	0

CEA, carcinoembryonic antigen; CI, confidence interval; CT, computed tomography; NSE, neuron-specific enolase; OR, odds ratio.

### 3.4 Model performance evaluation

**Figure 3:**
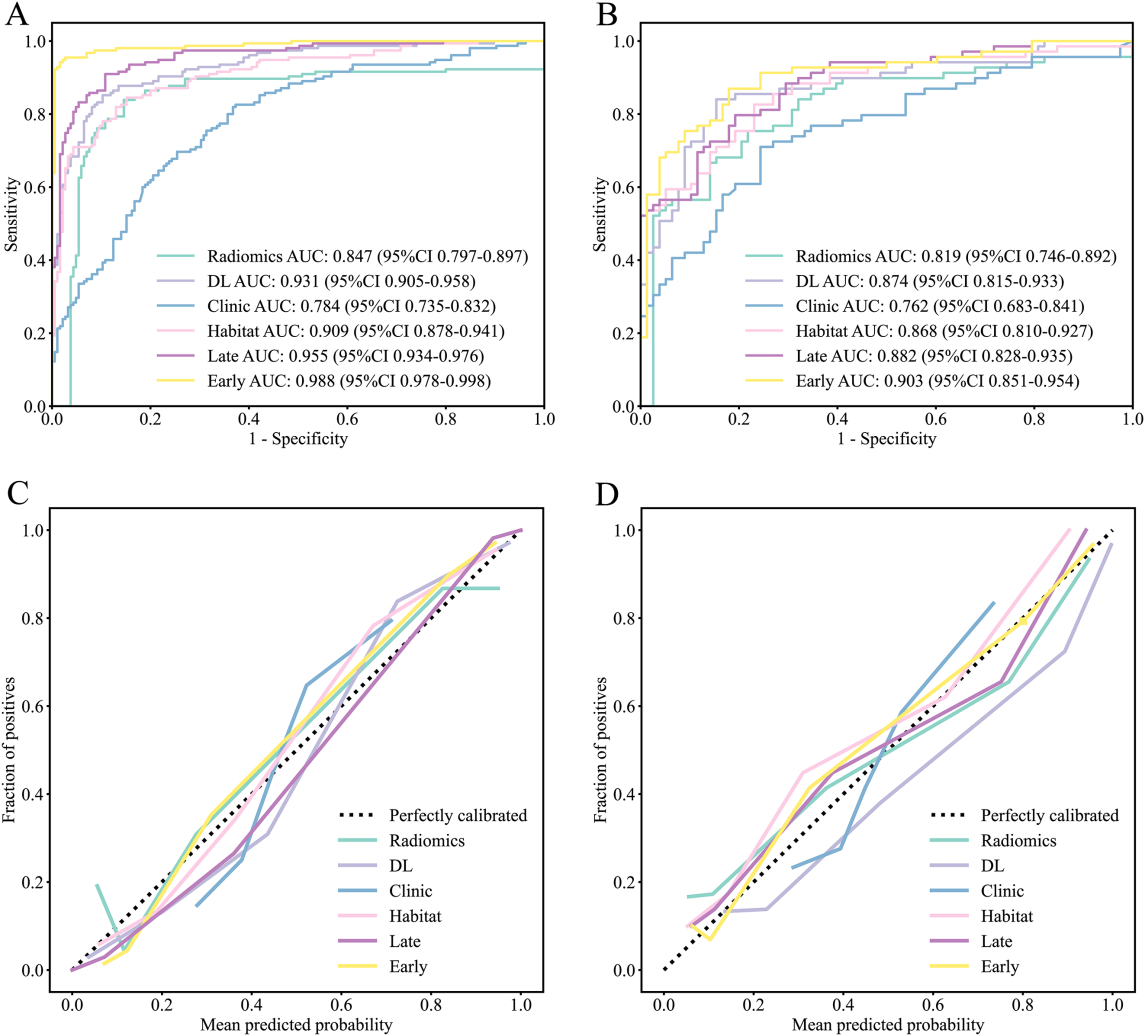
Receiver operating characteristic (ROC) curves and calibration curves of different models in the training cohort and the internal validation cohort. **(A)** ROC curves of different models in the training cohort. **(B)** ROC curves of different models in the internal validation cohort. **(C)** Calibration curves of different models in the training cohort. **(D)** Calibration curves of different models in the internal validation cohort. DL, deep learning.

**Figure 4:**
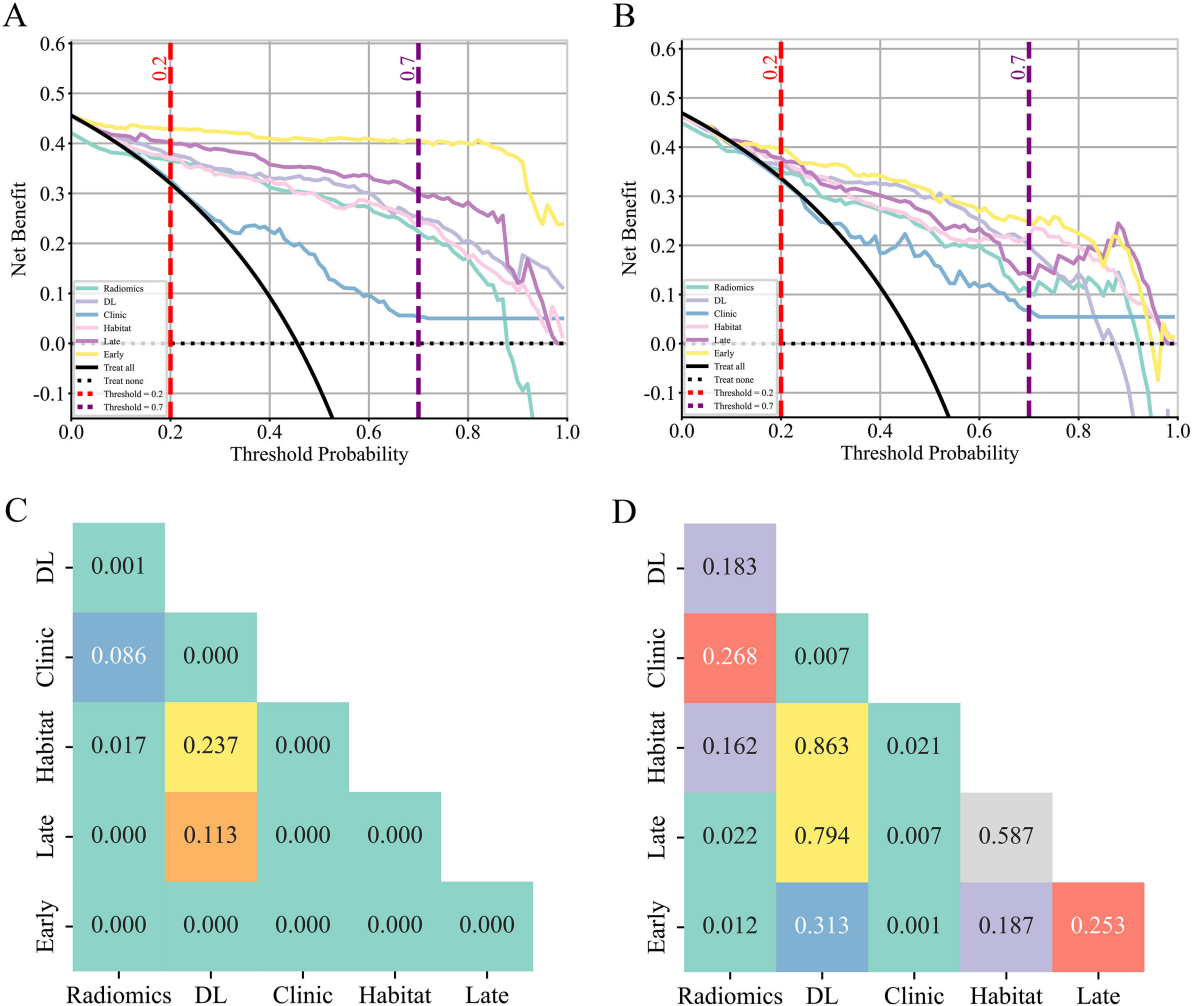
Decision curve analysis (DCA) and the DeLong test of different models in the training cohort and the internal validation cohort. **(A)** DCA of different models in the training cohort. **(B)** DCA of different models in the internal validation cohort. **(C)** Delong test of different models in the training cohort. **(D)** Delong test of different models in the internal validation cohort. DL, deep learning.

In the training cohort, the Early model achieved the highest AUC of 0.988 (95% CI 0.978–0.998), significantly outperforming all other models. In the internal validation cohort, the Early model maintained optimal performance with an AUC of 0.903 (95% CI 0.852–0.954). In the external test cohort, the Early model remained robust with an AUC of 0.872 (95% CI 0.810–0.934). DeLong test results further confirmed statistically significant AUC differences between the Early model and other models in the training cohort (p < 0.05) (**Table 3**; **Figures 3**, **4**; **Supplementary Figure 8**).

**Table 3:** Comparison of the performance of different models across three cohorts.

Model	AUC (95% CI)	Accuracy	Sensitivity	Specificity	PPV	NPV
Training Cohort						
Radiomics	0.847 (0.797–0.897)	0.847	0.839	0.854	0.828	0.863
DL	0.931 (0.905–0.958)	0.876	0.852	0.897	0.874	0.878
Clinic	0.784 (0.735–0.833)	0.718	0.826	0.627	0.650	0.811
Habitat	0.909 (0.878–0.941)	0.847	0.845	0.849	0.824	0.867
Late	0.955 (0.934–0.976)	0.900	0.910	0.892	0.876	0.922
Early	0.988 (0.978–0.998)	0.965	0.955	0.973	0.967	0.963
Internal Validation Cohort						
Radiomics	0.819 (0.746–0.892)	0.769	0.754	0.782	0.754	0.782
DL	0.874 (0.815–0.933)	0.844	0.841	0.846	0.829	0.857
Clinic	0.762 (0.683–0.841)	0.735	0.710	0.756	0.721	0.747
Habitat	0.868 (0.810–0.927)	0.796	0.826	0.769	0.760	0.833
Late	0.882 (0.828–0.935)	0.803	0.797	0.808	0.786	0.818
Early	0.903 (0.852–0.954)	0.844	0.870	0.821	0.811	0.877
External Test Cohort						
Radiomics	0.798 (0.709–0.887)	0.828	0.679	0.926	0.857	0.815
DL	0.834 (0.759–0.909)	0.821	0.774	0.852	0.774	0.852
Clinic	0.713 (0.626–0.801)	0.672	0.887	0.531	0.553	0.878
Habitat	0.823 (0.741–0.904)	0.828	0.717	0.901	0.826	0.830
Late	0.827 (0.744–0.910)	0.843	0.698	0.938	0.881	0.826
Early	0.872 (0.810–0.934)	0.828	0.830	0.827	0.759	0.882

AUC, area under the curve; CI, confidence interval; DL, deep learning; NPV, negative predictive value; PPV, positive predictive value.

The calibration curves were highly concordant with the predicted probabilities and observed incidence rates in the case of the Early model (Hosmer-Lemeshow test, p > 0.05 in all three cohorts), which means that this model has a good calibration (**Figure 3**; **Supplementary Figure 8**).

The DCA findings indicated that, within the clinically relevant threshold probability range of 0.20–0.70, the Early model achieved the highest net benefit in the training cohort, outperforming both the treat-all and treat-none strategies. A similar net-benefit advantage was observed for the Early model in the internal validation and external test cohorts across the same threshold range. (**Figure 4**; **Supplementary Figure 8**).

### 3.5 Model interpretation

**Figure 5:**
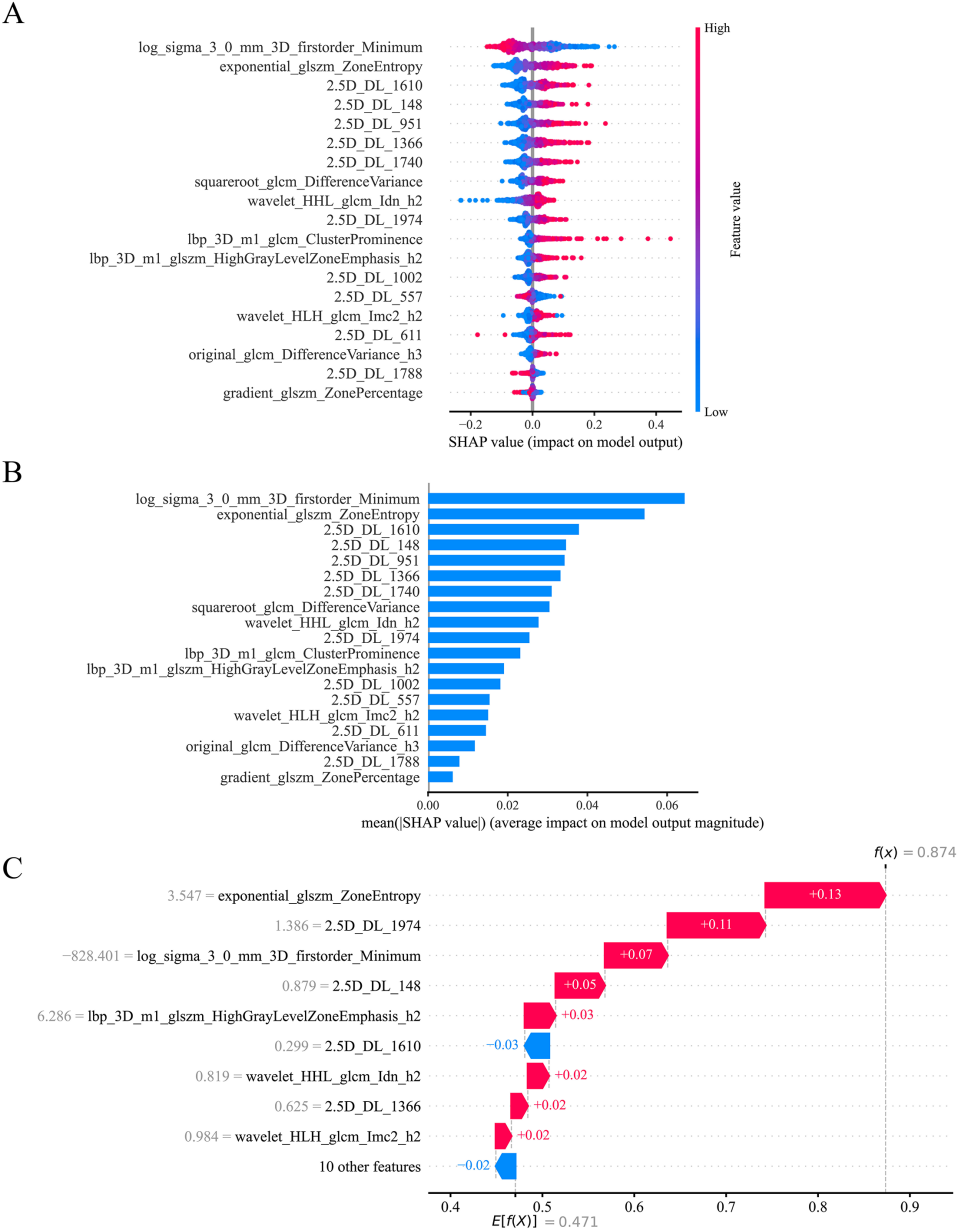
SHapley Additive exPlanations (SHAP)-based interpretation analysis of the early fusion model. **(A)** The SHAP summary plot. The plot visualizes the importance of global features and their impact on model predictions for the early fusion model. Features are ranked vertically by their contribution to the model output, with the top features having the greatest influence on distinguishing invasive adenocarcinoma. Each point represents a sample, where the horizontal position indicates the SHAP value (impact on model output), and the color represents the feature value (red for high, blue for low). For instance, “log_sigma_3_0_mm_3D_firstorder_Minimum” shows the highest importance, with high feature values (red dots) predominantly associated with negative SHAP values, indicating increased probability of adenocarcinoma in situ/minimally invasive adenocarcinoma prediction. This plot provides a comprehensive overview of which features drive the model’s decision-making process across the entire dataset. **(B)** The SHAP feature importance bar plot. Bar plots ranked features by mean absolute SHAP values, intuitively showing their average contribution magnitude to the model output and overall feature importance. **(C)** The SHAP waterfall plot. The plot demonstrates how individual features contribute to a specific prediction for a single sample. Starting from the base value E[f(X)] = 0.471 (the average model output across all samples), each horizontal bar shows how a particular feature pushes the prediction toward the final output f(x) = 0.874. Red bars represent features that increase the prediction value, while blue bars represent features that decrease it. For this particular case, “exponential_glszm_ZoneEntropy” contributes the most (+0.13), followed by “2.5D_DL_1974” (+0.11), while “2.5D_DL_1610” has a negative contribution (−0.03). The cumulative effect of all features moves the prediction from the baseline to the final value, providing transparent, case-specific model interpretation that can support clinical decision-making. SHAP, SHapley Additive exPlanations.

We used SHAP analysis to interpret the Early model quantitatively. Summary plots intuitively showed contributions of each feature (ranked by significance) to the model’s output, and the scatter distribution indicated the direction of influence of feature values, providing overall model interpretation (**Figure 5A**). Bar plots intuitively ranked features by their mean absolute SHAP values, highlighting the overall importance of each feature and its average contribution magnitude to the model output (**Figure 5B**). Waterfall plots intuitively showed the way each feature drove the output of the model, starting at the baseline value to the final value predicted per individual sample, and made it clear that feature contribution magnitude and impact direction were different (**Figure 5C**).

## 4 Discussion

The proposed model of this work is a multi-scale fusion model combining radiomics, habitat, DL, and clinical information to obtain accurate preoperative differentiation of invasion grade (AIS/MIA versus IAC) in lung adenocarcinoma-related GGNs. As shown by the results, the Early model showed better diagnostic outcomes in all three cohorts, far surpassing single-scale models. The result gives a new technical route to precise preoperative diagnosis of GGNs, which helps optimize surgical plans and prevents under-treatment or over-treatment.

The presence of intratumoral heterogeneity is an essential biological feature of malignant tumors, which are strongly linked to tumor invasiveness, therapy responsiveness, and prognosis (23). In this research, K-means clustering was used to divide GGNs into three subregions, a classification that had some pathophysiological basis. Imaging features of GGNs actually capture the variation in alveolar structure preservation, tumor cell density distribution, and interstitial fibrosis stages (3). Upon development of AIS into IAC, tumor cells gradually infiltrate basement membranes and infiltrate nearby stroma, which leads to intratumoral increases in solid components as well as changes in the density level of vasculature and the process of stromal remodeling (2, 4, 6). Namely, areas of high-density habitat subregions can be related to invasive regions of dense tumor cells with significant interstitial fibrosis, and areas of low-density habitat subregions can be indicative of non-invasive regions where lepidic tumor cell growth occurs and alveolar structures are relatively intact, and areas of moderate-density habitat subregions can be transitional areas of tumor invasion where there is partial destruction of alveolar structures and interstitial proliferation (3, 4). Such habitat partitioning based on images has a high correlation with the heterogeneity of tumors in terms of their histopathology (14). The Habitat models demonstrated AUCs of 0.909, 0.868, and 0.823 across three cohorts, which were higher than those of the Radiomics models (0.847, 0.819, and 0.798). The result is consistent with other studies (17, 24), suggesting that the habitat analysis, by its ability to assess intratumoral spatial heterogeneity, may add value to the diagnostic and prognosis prediction of diseases.

The use of DL has also been proven to have great potential in medical image analysis. This paper systematically built and compared nine DL models depending on 2D, 2.5D, and 3D input modes and three network architectures (ResNet18, ResNet50, and ResNet101). The results revealed that the 2.5D ResNet50 model had the best AUC (0.874) in the internal validation cohort, which is higher than 2D and 3D models. In contrast to 2D models, 2.5D models combine data of the maximum lesion slice with the adjacent superior and inferior slices, which can represent some three-dimensional spatial relationships and more accurately reflect the three-dimensional morphology of the lesion and its surrounding tissues (25). Yang et al. also demonstrated the same fact that the 2.5D model outperforms 2D methods in the estimation of the pathological grading of renal clear cell carcinoma (26). It is interesting to note that the 3D deep learning models’ performance did not surpass 2D and 2.5D models as anticipated. There are a number of reasons behind it. High-quality 3D pre-trained models within the medical field are still quite scarce; even though Med3D was used to pre-train in this study, its domain adaptability should be improved. Also, 3D models have significantly more parameters than their 2D and 2.5D equivalents, making them more prone to performance degradation when trained on small amounts of data (27). Moreover, the diagnostic-relevant information in GGNs might be mostly localized in particular slices, so that adding the whole three-dimensional volume would add redundant noise instead of an extra discriminative signal. In terms of network architecture, ResNet50 performed better than both ResNet18 and ResNet101, presumably because it strikes the right balance between model complexity and generalization ability. With its shallow structure, ResNet18 shows low feature extraction ability, and ResNet101 is hard to train because it is overparameterized (28).

Multi-scale information fusion represents an important strategy for enhancing diagnostic performance. This study systematically compared early fusion (feature-level fusion) and late fusion (decision-level fusion) strategies. Results showed early fusion outperformed late fusion across all three cohorts. Feature-level fusion allows sufficient interaction and synergy among different scale features, enabling models to learn more complex and refined feature combination patterns (29). For example, radiomics features may complement DL features—the former excelling at capturing hand-crafted texture and shape features while the latter automatically learns high-level abstract features. Concurrently, through LASSO feature selection, the Early model automatically identifies and retains the most discriminative feature combinations, achieving an optimal balance between dimensionality reduction and information preservation. Recent studies also support early fusion advantages (29, 30). Nevertheless, this apparent advantage should be interpreted with caution. We observed a stepwise decrease in AUC for the Early model from the training cohort (0.988) to internal validation (0.903) and external testing (0.872), which suggests model optimism and potential overfitting despite LASSO-based reduction. In our setting, 19 features were selected from thousands of candidates using 340 training samples; under such high-dimensional, relatively small-sample conditions, feature selection may remain unstable and the apparent training AUC may be inflated. Therefore, the training AUC is better viewed as an upper-bound estimate rather than a reliable indicator of real-world performance, whereas the validation and especially external-test results are more representative of generalizability. This performance gap may also reflect inter-cohort heterogeneity, including differences in imaging protocols, patient characteristics, and data noise. Future work should focus on larger multicenter cohorts, stronger harmonization strategies, and more conservative resampling frameworks to improve robustness. Fusion strategy selection should be optimized according to specific application scenarios and data characteristics; late fusion may demonstrate advantages in certain situations, particularly when base models have high feature correlation with redundant information (31).

The SHAP analysis findings support the biological and radiological rationale underlying the Early model. The highest-contributing radiomics feature was log_sigma_3_0_mm_3D_firstorder_Minimum, which represents the minimum first-order intensity value extracted from three-dimensional images after Laplacian of Gaussian (LoG, σ = 3.0 mm) filtering, and captures the most pronounced low-gray regions within the lesion (32). In the present study, SHAP analysis revealed that lower values of this feature were associated with higher model outputs, suggesting that lesions harboring more conspicuous extremely low-gray components were more likely to be classified as IAC. Such low-gray phenotypes may correspond to underlying histological substrates, including necrosis, cystic degeneration, or focal hypoperfusion, reflecting impaired oxygen supply and a deteriorating tumor microenvironment (33, 34). A prior study (35) demonstrated that radiomics features capable of characterizing marked intratumoral gray heterogeneity and focal architectural disruption are frequently associated with greater invasiveness and poorer prognosis, findings consistent with those of the present study. The presence of multiple highly ranked 2.5D DL features (2.5D_DL_1610, 2.5D_DL_148, 2.5D_DL_951, etc.) indicates that the contextual information from the largest cross-section of the tumor and its adjacent layers contains important clues related to invasion. Although the human eye cannot directly interpret these potential features, they may encode complex combinations such as morphology, texture, and spatial context. Habitat features also account for a certain proportion in the SHAP ranking, indicating that tumor internal heterogeneity is of great value in assessing invasiveness. By quantifying distinct imaging subregions and their spatial distributions, these features can indirectly reflect microenvironmental differences in cell density, fibrosis, and stromal remodeling (3, 19). Taken together, these findings indicate that the Early model does not rely on a single feature category, but rather integrates global lesion features, intratumoral spatial heterogeneity, and deep contextual image information. This multi-scale feature representation is clinically reasonable and provides an interpretable explanation for the improved discrimination between AIS/MIA and IAC.

This study has the following limitations: First, as a retrospective study, selection bias may exist. Although strict inclusion and exclusion criteria were applied, the inherent limitations of retrospective design cannot be eliminated. Future prospective multicenter studies are needed to further validate clinical utility. Second, although we systematically compared multiple DL architectures, many emerging architectures (e.g., Vision Transformer, Swin Transformer, etc.) were not included. With rapid DL advancements, continuous model architecture updates and optimization are necessary. Third, this study did not incorporate proteomic or genomic data; with precision medicine development, multi-omics information fusion may further enhance predictive performance. Fourth, as this was a retrospective study, routine pathological processing did not include specimen-oriented sectioning or radiology-pathology spatial co-registration; thus, the pathological interpretation of the three habitat subregions remains speculative. Future studies integrating imaging-pathology registration are warranted to clarify the biological significance of each habitat subregion.

The multi-scale fusion model constructed in this study integrates radiomics, habitat, DL, and clinical information, enabling precise preoperative differentiation between AIS/MIA and IAC with excellent diagnostic performance and favorable clinical application potential. This comprehensive study provides an innovative solution for precise GGNs diagnosis and individualized treatment decision-making, potentially advancing clinical translation of artificial intelligence-assisted diagnostic technology in thoracic imaging.

## Data Availability

The raw data supporting the conclusions of this article will be made available by the authors, without undue reservation.

## References

[1] Bray F Laversanne M Sung H Ferlay J Siegel RL Soerjomataram I . Global cancer statistics 2022: globocan estimates of incidence and mortality worldwide for 36 cancers in 185 countries. Ca: A Cancer J For Clin. (2024) 74:229–63. doi: 10.3322/caac.21834. PMID: 38572751

[2] Fu B Lv F Li W Lin R Zheng Y Chu Z . Significance of intra-nodular vessel sign in differentiating benign and Malignant pulmonary ground-glass nodules. Insights Imaging. (2021) 12:65. doi: 10.1186/s13244-021-01012-7. PMID: 34037864 PMC8155149

[3] Nicholson AG Tsao MS Beasley MB Borczuk AC Brambilla E Cooper WA . The 2021 who classification of lung tumors: impact of advances since 2015. J Thorac Oncol. (2022) 17:362. doi: 10.1016/j.jtho.2021.11.003. PMID: 34808341

[4] Yotsukura M Asamura H Motoi N Kashima J Yoshida Y Nakagawa K . Long-term prognosis of patients with resected adenocarcinoma in situ and minimally invasive adenocarcinoma of the lung. J Thorac Oncol. (2021) 16:1312–20. doi: 10.1016/j.jtho.2021.04.007. PMID: 33915249

[5] Tsutani Y Miyata Y Nakayama H Okumura S Adachi S Yoshimura M . Appropriate sublobar resection choice for ground glass opacity-dominant clinical stage ia lung adenocarcinoma: wedge resection or segmentectomy. Chest. (2014) 145:66. doi: 10.1378/chest.13-1094. PMID: 24551879

[6] Fan L Fang M Li Z Tu W Wang S Chen W . Radiomics signature: a biomarker for the preoperative discrimination of lung invasive adenocarcinoma manifesting as a ground-glass nodule. Eur Radiol. (2019) 29:889–97. doi: 10.1007/s00330-018-5530-z. PMID: 29967956

[7] Travis WD Brambilla E Noguchi M Nicholson AG Geisinger KR Yatabe Y . International association for the study of lung cancer/american thoracic society/european respiratory society international multidisciplinary classification of lung adenocarcinoma. J Thorac Oncol. (2011) 6:244–85. doi: 10.1097/JTO.0b013e318206a221. PMID: 21252716 PMC4513953

[8] Zwanenburg A Vallières M Abdalah MA Aerts HJWL Andrearczyk V Apte A . The image biomarker standardization initiative: standardized quantitative radiomics for high-throughput image-based phenotyping. Radiology. (2020) 295:328–38. doi: 10.1148/radiol.2020191145. PMID: 32154773 PMC7193906

[9] Sun Q Chen Y Liang C Zhao Y Lv X Zou Y . Biologic pathways underlying prognostic radiomics phenotypes from paired mri and rna sequencing in glioblastoma. Radiology. (2021) 301:654–63. doi: 10.1148/radiol.2021203281. PMID: 34519578

[10] Li ZC Bai H Sun Q Zhao Y Lv Y Zhou J . Multiregional radiomics profiling from multiparametric mri: identifying an imaging predictor of idh1 mutation status in glioblastoma. Cancer Med. (2018) 7:5999–6009. doi: 10.1002/cam4.1863. PMID: 30426720 PMC6308047

[11] Yan J Liu L Wang W Zhao Y Li KK Li K . Radiomic features from multi-parameter mri combined with clinical parameters predict molecular subgroups in patients with medulloblastoma. Front Oncol. (2020) 10:558162. doi: 10.3389/fonc.2020.558162. PMID: 33117690 PMC7566191

[12] Zhao Y Liu G Sun Q Zhai G Wu G Li Z . Validation of ct radiomics for prediction of distant metastasis after surgical resection in patients with clear cell renal cell carcinoma: exploring the underlying signaling pathways. Eur Radiol. (2021) 31:5032–40. doi: 10.1007/s00330-020-07590-2. PMID: 33439312

[13] Yan J Zhao Y Chen Y Wang W Duan W Wang L . Deep learning features from diffusion tensor imaging improve glioma stratification and identify risk groups with distinct molecular pathway activities. Ebiomedicine. (2021) 72:103583. doi: 10.1016/j.ebiom.2021.103583. PMID: 34563923 PMC8479635

[14] Gao Y Li Z Zhai X Zhang G Zhang L Huang T . Mri-based habitat radiomics combined with vision transformer for identifying vulnerable intracranial atherosclerotic plaques and predicting stroke events: a multicenter, retrospective study. Eclinicalmedicine. (2025) 82:103186. doi: 10.1016/j.eclinm.2025.103186. PMID: 40235946 PMC11999680

[15] Dong N Wei S Zheng L Huang D Zhang G Li Y . Nomogram integrating clinical-radiological and radiomics features for differentiating invasive from non-invasive pulmonary adenocarcinomas presenting as ground-glass nodules. Am J Cancer Res. (2025) 15:797–810. doi: 10.62347/AOAN9966. PMID: 40084360 PMC11897637

[16] Feng H Shi G Xu Q Ren J Wang L Cai X . Radiomics-based analysis of ct imaging for the preoperative prediction of invasiveness in pure ground-glass nodule lung adenocarcinomas. Insights Imaging. (2023) 14:24. doi: 10.1186/s13244-022-01363-9. PMID: 36735104 PMC9898484

[17] Wang Q Zhang Y Wang T Chen Y Yan R Liu K . Mri-based habitat analysis for the prediction of progression-free survival in primary spinal tumors. Radiology. (2025) 317:e242993. doi: 10.1148/radiol.242993. PMID: 41147907 PMC12767461

[18] Dong N Yan Y Li Y Li G Wang P Li L . Ct-based habitat radiomics for preoperative differentiation of adenocarcinoma in situ/minimally invasive adenocarcinoma from invasive adenocarcinoma manifesting as ground-glass nodules: a multicenter study. Front Oncol. (2025) 15:1660071. doi: 10.3389/fonc.2025.1660071. PMID: 41170450 PMC12568381

[19] Shang Y Zeng Y Luo S Wang Y Yao J Li M . Habitat imaging with tumoral and peritumoral radiomics for prediction of lung adenocarcinoma invasiveness on preoperative chest ct: a multicenter study. Ajr Am J Roentgenol. (2024) 223:e2431675. doi: 10.2214/AJR.24.31675. PMID: 39140631

[20] Du H Shen J Chen F Wang K Qin L Hu Y . Integrating ct-based radiomics and deep learning for invasive prediction of ground-glass nodules in lung adenocarcinoma: a multicohort study. Insights Imaging. (2025) 16:271. doi: 10.1186/s13244-025-02156-6. PMID: 41359094 PMC12686268

[21] Fu CL Yang ZB Li P Shan KF Wu MK Xu JP . Discrimination of ground‐glass nodular lung adenocarcinoma pathological subtypes via transfer learning: a multicenter study. Cancer Med. (2023) 12:18460–9. doi: 10.1002/cam4.6402. PMID: 37723872 PMC10557850

[22] Orlhac F Frouin F Nioche C Ayache N Buvat I . Validation of a method to compensate multicenter effects affecting ct radiomics. Radiology. (2019) 291:53–9. doi: 10.1148/radiol.2019182023. PMID: 30694160

[23] Vitale I Shema E Loi S Galluzzi L . Intratumoral heterogeneity in cancer progression and response to immunotherapy. Nat Med. (2021) 27:212–24. doi: 10.1038/s41591-021-01233-9. PMID: 33574607

[24] Chen H Liu Y Zhao J Jia X Chai F Peng Y . Quantification of intratumoral heterogeneity using habitat-based mri radiomics to identify her2-positive, -low and -zero breast cancers: a multicenter study. Breast Cancer Res. (2024) 26:160. doi: 10.1186/s13058-024-01921-7. PMID: 39578913 PMC11583526

[25] Zhu J Xu B Fan T Ji S Gu K Ding J . Sub-regional radiomics combining multichannel 2-dimensional or 3-dimensional deep learning for predicting neoadjuvant chemo-immunotherapy response in esophageal squamous cell carcinoma: a multicenter study. NPJ Precis Oncol. (2025) 9:248. doi: 10.1038/s41698-025-01047-9. PMID: 40691312 PMC12279934

[26] Yang Z Jiang H Shan S Wang X Kou Q Wang C . 2.5d deep learning-based prediction of pathological grading of clear cell renal cell carcinoma using contrast-enhanced ct: a multicenter study. Acad Radiol. (2025) 32:5907–16. doi: 10.1016/j.acra.2025.06.056. PMID: 40683765

[27] Zhang W Zhao X Meng L Lu L Guo J Cheng M . A multicenter comparative analysis of radiomics, deep-learning, and fusion models for predicting postpartum hemorrhage. Acad Radiol. (2025) 32:5930–9. doi: 10.1016/j.acra.2025.05.068. PMID: 40562675

[28] He K Zhang X Ren S Sun J . (2016). “ Deep residual learning for image recognition”, in: Proceedings of the Ieee Conference On Computer Vision and Pattern Recognition, 770–8.

[29] Hyun SH Ahn MS Koh YW Lee SJ . A machine-learning approach using pet-based radiomics to predict the histological subtypes of lung cancer. Clin Nucl Med. (2019) 44:956–60. doi: 10.1097/RLU.0000000000002810. PMID: 31689276

[30] Liu J Chen Y Lan L Lin B Chen W Wang M . Prediction of rupture risk in anterior communicating artery aneurysms with a feed-forward artificial neural network. Eur Radiol. (2018) 28:3268–75. doi: 10.1007/s00330-017-5300-3. PMID: 29476219

[31] Wang W Liang H Zhang Z Xu C Wei D Li W . Comparing three-dimensional and two-dimensional deep-learning, radiomics, and fusion models for predicting occult lymph node metastasis in laryngeal squamous cell carcinoma based on ct imaging: a multicenter, retrospective, diagnostic study. Eclinicalmedicine. (2024) 67:102385. doi: 10.1016/j.eclinm.2023.102385. PMID: 38261897 PMC10796944

[32] van Griethuysen JJM Fedorov A Parmar C Hosny A Aucoin N Narayan V . Computational radiomics system to decode the radiographic phenotype. Cancer Res. (2017) 77:e104–7. doi: 10.1158/0008-5472.CAN-17-0339. PMID: 29092951 PMC5672828

[33] Lubner MG Smith AD Sandrasegaran K Sahani DV Pickhardt PJ . Ct texture analysis: definitions, applications, biologic correlates, and challenges. Radiographics. (2017) 37:1483–503. doi: 10.1148/rg.2017170056. PMID: 28898189

[34] Höckel M Vaupel P . Tumor hypoxia: definitions and current clinical, biologic, and molecular aspects. J Natl Cancer Institute. (2001) 93:266–76. doi: 10.1093/jnci/93.4.266. PMID: 11181773

[35] Cook GJR Yip C Siddique M Goh V Chicklore S Roy A . Are pretreatment 18f-fdg pet tumor textural features in non-small cell lung cancer associated with response and survival after chemoradiotherapy? J Nucl Medicine: Off Publication Soc Nucl Med. (2013) 54:19–26. doi: 10.2967/jnumed.112.107375. PMID: 23204495

