# Supplementary material for: A CT-based multi-scale fusion model with SHAP interpretation for preoperative differentiation between lung adenocarcinoma in situ/minimally invasive adenocarcinoma and invasive adenocarcinoma: a multicenter study
Source: Front Oncol. 2026 May 12;16:1824735. doi: 10.3389/fonc.2026.1824735 (PMC13201193; doi:10.3389/fonc.2026.1824735)
Supplement: Supplementary file 1 [file DataSheet1.docx]

Supplementary Material

# Supplementary S1. Inclusion and exclusion criteria

Inclusion criteria: (1) postoperative histopathological confirmation of adenocarcinoma in situ, minimally invasive adenocarcinoma, or invasive adenocarcinoma; (2) GGNs with a long diameter of less than 30 mm; (3) availability of a chest CT scan performed within two weeks prior to surgery.

Exclusion criteria: (1) incomplete clinical, imaging, or pathological data; (2) poor-quality chest CT images (e.g., significant metal artifacts, respiratory motion artifacts) or a slice thickness greater than 2 mm; (3) presence of multiple GGNs within the same pulmonary lobe; (4) presence of a concurrent malignancy in other organs; (5) any form of antitumor therapy before surgery, including but not limited to radiotherapy, chemotherapy, or immunotherapy.

# Supplementary S2. Habitat generation

Step1. Local features extraction

A 3 × 3 × 3 sliding cubic window was used to traverse the ground-glass nodules (GGNs), and 19-dimensional local features were calculated for each voxel (Figure S1). This strategy constructs a high-dimensional discriminative space through multi-scale and multi-directional fine characterization, providing a robust data foundation for subsequent precise subregion delineation.

Step2. K-means subregion clustering

K-means is a distance-based clustering method whose core principle is to partition n objects into k clusters so that objects within the same cluster are as compact as possible. For each voxel within the GGNs, the Euclidean distance between its 19-dimensional feature vector and each cluster center was calculated, and the voxel was assigned to the corresponding cluster based on the nearest distance criterion. Through iterative updates of cluster centers, k subregions were ultimately obtained within the GGNs. The objective function of the K-means algorithm can be expressed as:

$$J=\sum_{i=1}^{K} \sum_{j=1}^{n} \left\| x_{j}-\mu_{i} \right\|^{2}$$

Where：

- *J* is the sum of squared errors within clusters.
- *K* is the number of clusters.
- *n* is the total number of data points.
- $x_{j}$ is the j-th point in the dataset.
- $\mu_{i}$ is the center of the i-th cluster.

By iterating through k = 2–9 and selecting the value that maximized the Calinski-Harabasz index as the optimal number of clusters, we ensured that each subregion possessed optimal separability and internal consistency in the 19-dimensional feature space, providing reliable functional subregions for subsequent habitat analysis.

# Supplementary S3. Extraction and selection of radiomics and habitat features

This study strictly followed the Imaging Biomarker Standardization Initiative (IBSI) guidelines, extracting radiomics features from the tumor region of interest (ROI) and habitat subregions using the PyRadiomics platform (version 3.0.1). Extracted features encompassed three major categories: (1) first-order histogram features; (2) shape features; (3) texture features, specifically including Gray Level Co-occurrence Matrix, Gray Level Dependence Matrix, Gray Level Run Length Matrix, Gray Level Size Zone Matrix, and Neighboring Gray Tone Difference Matrix.

To improve the reproducibility of feature extraction and enhance model generalization, this study randomly selected 30 GGNs, each independently delineated by radiologists A and B. The interclass correlation coefficient was used to assess inter-observer variability. After 2 weeks, radiologist A performed a second independent delineation of the same nodules, and the intraclass correlation coefficient was calculated to evaluate intra-observer variability. Ultimately, only highly stable features with both intraclass and interclass correlation coefficients greater than 0.75 were retained for subsequent analysis and modeling. Given that clustering itself is an unsupervised method, this step was not executed for habitat feature selection.

To eliminate batch effects arising from differences in data acquisition equipment and scanning parameters across two centers, ComBat harmonization was applied to the radiomics features to achieve cross-center data calibration. All features were then Z-score standardized using the mean and standard deviation of the training cohort to eliminate dimensional differences. Pearson correlation coefficients were calculated to remove highly redundant variables with values ≥ 0.9. The minimum Redundancy Maximum Relevance algorithm selected the top 30 features with high target variable correlation and low redundancy. Finally, the least absolute shrinkage and selection operator regression model was fit to the training cohort to shrink non-important feature coefficients to zero automatically. 10-fold cross-validation selected the optimal λ value, retaining features with non-zero coefficients to achieve dimensionality reduction, key information preservation, and enhanced model interpretability and predictive performance.

# Supplementary S4. Deep learning model training

⚫ **2D deep learning**

In the 2D deep learning model, the bounding box is a rectangular region delineating the maximum tumor cross-section, used to crop the largest tumor ROI. After image cropping, linear interpolation was employed to resize the images to 224 × 224 pixels. Z-score normalization was applied to the intensity distribution of the RGB channels, standardizing pixel values to follow a standard normal distribution. Real-time data augmentation was implemented during the training phase, including random cropping, horizontal flipping, and vertical flipping; only normalization was performed during the testing phase.

We adopted a transfer learning strategy, initializing the model with ImageNet pre-trained weights to enhance its cross-domain adaptation capability. Learning rate adjustment is crucial for generalization performance; therefore, a cosine annealing strategy was selected, formulated as follows:

$$\eta_{t}=\eta_{min}^{i}+\frac{1}{2}\left( \eta_{max}^{i}-\eta_{min}^{i} \right)\left( 1+\cos\left( \frac{T_{\mathrm{cur}}}{T_{i}}\pi\right) \right)$$

where $\eta_{min}^{i}$ = 0 is the minimum learning rate, $\eta_{max}^{i}$ = 0.01 is the maximum learning rate, and $T_{i}$ = 50 is the number of epochs. Other key hyperparameters included: stochastic gradient descent (SGD) as the optimizer, softmax cross-entropy as the loss function, and a batch size of 32.

⚫ **2.5D deep learning**

For each pulmonary nodule, we first obtained three consecutive slices by cropping the largest tumor ROI on the axial plane, along with one slice above and one slice below. Subsequently, these three slices were uniformly resized to 224 × 224 pixels, and pixel values were normalized to standardize the intensity distribution. Finally, these three slice images were fused to form the 2.5D image input. To enhance training dataset diversity and improve model robustness, real-time data augmentation techniques were applied during the training phase, including random cropping, horizontal flipping, and vertical flipping. Hyperparameter settings remained consistent with the 2D deep learning model.

⚫ **3D deep learning**

To overcome the scarcity of medical three-dimensional pre-trained models, this study employed the multi-modal, multi-organ Med3D dataset as a pre-training resource. We utilized the Med3D pre-trained 3D-ResNet as the backbone network and constructed a specialized architecture for invasive adenocarcinoma prediction by replacing the original decoder with fully connected layers. The backbone was initialized with pre-trained weights and fine-tuned on our training dataset to achieve domain adaptation. In 3D deep learning, the bounding box refers to the minimum enclosing cube of the tumor ROI. Linear interpolation was used to resize the cube to 96 × 96 × 96 voxels. To augment the dataset, data augmentation strategies, including flipping along the x-, y-, and z-axes, were introduced. The ROI cube was then used as input to the 3D deep learning model. Training was conducted for 300 epochs, with other hyperparameters remaining consistent with the 2D and 2.5D models.

# Supplementary S5. The algorithm type, feature sampling strategy, and calculation parameters of the SHapley Additive exPlanations (SHAP) analysis

# ⚫ SHAP algorithm type

The final early fusion model was a binary support vector machine (SVM) with a radial basis function (RBF) kernel. Because this classifier is a non-linear and non-tree-based model, we used the model-agnostic Kernel SHAP method for interpretation, rather than Tree SHAP or Linear SHAP. Kernel SHAP estimates Shapley values by approximating the contribution of each feature to the model prediction through a locally weighted surrogate model, and is therefore appropriate for explaining complex non-linear classifiers such as the present SVM model.

⚫ **Feature sampling strategy**

The SHAP analysis was conducted on the **19 non-zero features** in the early fusion model. **No additional feature subsampling was applied at the SHAP stage**; thus, all 19 features were included simultaneously in the interpretation analysis.

For the background/reference distribution required by Kernel SHAP, the entire training dataset was not used directly because of the high computational burden associated with non-linear models. Instead, the selected training data were summarized using **k-means clustering into 50 representative centroids**, and these centroids served as the SHAP background dataset. This approach preserves the overall distributional characteristics of the training cohort while reducing computational complexity and improving estimation stability. In Kernel SHAP, these background samples are used during feature masking and coalition construction, and therefore define the reference distribution for estimating feature contributions.

⚫ **SHAP calculation parameters**

SHAP values were calculated based on the model output on the probability scale, with the positive class defined as Invasive adenocarcinoma (IAC). Accordingly, each SHAP value represents the contribution of a feature to increasing or decreasing the predicted probability of IAC.

The coalition sampling parameter was not manually specified and therefore followed the default automatic sampling setting of the SHAP KernelExplainer, under which the number of model evaluations is determined automatically according to the dimensionality of the input feature space. For the 19 features in the early fusion model, this corresponded to approximately 2086 model evaluations per explained sample. All other parameters that were not explicitly specified in our implementation followed the default settings of the SHAP Python package.

# Supplementary Figures and Tables

## Supplementary Tables

Table S1. Specific parameters of CT scanners at different centers

| Settings | Center 1 | |  | Center 2 | |
| --- | --- | --- | --- | --- | --- |
|  | GE Revolution | Philips Incisive |  | Philips Brilliance | GE LightSpeed VCT |
| Detector rows | 256 | 64 |  | 128 | 64 |
| Tube voltage (kV) | 120 | 120 |  | 120 | 120 |
| Tube current (mAs) | 100–500  (automatically optimized) | 50–350  (automatically optimized) |  | 50–300  (automatically optimized) | 50–400  (automatically optimized) |
| Pitch | 0.984 | 1.000 |  | 0.797 | 0.984 |
| Collimation (mm) | 256 × 0.625 | 64 × 0.625 |  | 128 × 0.625 | 64 × 0.625 |
| Matrix size | 512 × 512 | 512 × 512 |  | 512 × 512 | 512 × 512 |
| Field of view (cm) | 40 | 40 |  | 40 | 40 |
| Reconstruction thickness (mm) | 1.25 | 1 |  | 1 | 1.25 |
| Reconstruction interval (mm) | 1 | 1 |  | 1 | 1.25 |
| Reconstruction algorithm | Standard | Standard |  | Standard | Standard |

Table S2. Baseline features in the training cohort, internal validation cohort, and external test cohort

| Features | Training Cohort  (n = 340) | Internal Validation Cohort  (n = 147) | External Test Cohort  (n = 134) | P |
| --- | --- | --- | --- | --- |
| Gender [n (%)] |  |  |  | 0.643 |
| Male | 111 (32.65) | 42 (28.57) | 44 (32.84) |  |
| Female | 229 (67.35) | 105 (71.43) | 90 (67.16) |  |
| Smoking [n (%)] |  |  |  | 0.948 |
| No | 297 (87.35) | 127 (86.39) | 116 (86.57) |  |
| Yes | 43 (12.65) | 20 (13.61) | 18 (13.43) |  |
| Location [n (%)] |  |  |  | 0.837 |
| RUL | 106 (31.18) | 47 (31.97) | 44 (32.84) |  |
| RML | 20 (5.88) | 10 (6.80) | 10 (7.46) |  |
| RLL | 57 (16.76) | 29 (19.73) | 23 (17.16) |  |
| LUL | 97 (28.53) | 39 (26.53) | 42 (31.34) |  |
| LLL | 60 (17.65) | 22 (14.97) | 15 (11.19) |  |
| Lobulation [n (%)] |  |  |  | 0.565 |
| No | 77 (22.65) | 39 (26.53) | 35 (26.12) |  |
| Yes | 263 (77.35) | 108 (73.47) | 99 (73.88) |  |
| Spiculation [n (%)] |  |  |  | 0.074 |
| No | 218 (64.12) | 85 (57.82) | 95 (70.90) |  |
| Yes | 122 (35.88) | 62 (42.18) | 39 (29.10) |  |
| Margin [n (%)] |  |  |  | 0.789 |
| Clear | 217 (63.82) | 89 (60.54) | 84 (62.69) |  |
| Unclear | 123 (36.18) | 58 (39.46) | 50 (37.31) |  |
| Vessel changes [n (%)] |  |  |  | 0.915 |
| No | 115 (33.82) | 50 (34.01) | 48 (35.82) |  |
| Yes | 225 (66.18) | 97 (65.99) | 86 (64.18) |  |
| Bubble lucency [n (%)] |  |  |  | 0.800 |
| No | 236 (69.41) | 102 (69.39) | 97 (72.39) |  |
| Yes | 104 (30.59) | 45 (30.61) | 37 (27.61) |  |
| Pleural retraction [n (%)] |  |  |  | 0.250 |
| No | 210 (61.76) | 81 (55.10) | 86 (64.18) |  |
| Yes | 130 (38.24) | 66 (44.90) | 48 (35.82) |  |
| Shape [n (%)] |  |  |  | 0.300 |
| Round | 142 (41.76) | 64 (43.54) | 47 (35.07) |  |
| Irregular | 198 (58.24) | 83 (56.46) | 87 (64.93) |  |
| Long diameter (mm) | 12.17 ± 5.56 | 11.68 ± 5.12 | 11.92 ± 5.88 | 0.655 |
| Short diameter (mm) | 9.13 ± 3.84 | 8.88 ± 3.82 | 8.31 ± 3.51 | 0.105 |
| Age (years) | 55.28 ± 11.77 | 57.33 ± 10.94 | 55.75 ± 11.02 | 0.190 |
| NSE (ng/ml) | 15.08 ± 5.12 | 15.05 ± 5.55 | 14.89 ± 4.39 | 0.930 |
| CEA (ng/ml) | 1.92 ± 1.24 | 1.97 ± 1.32 | 2.45 ± 8.23 | 0.415 |
| CT value (HU) | -418.98 ± 196.69 | -401.39 ± 198.86 | -411.72 ± 185.46 | 0.655 |

CEA, carcinoembryonic antigen; CT, computed tomography; HU, Hounsfield units; LLL, left lower lobe; LUL, left upper lobe; NSE, neuron-specific enolase; RLL, right lower lobe; RML, right middle lobe; RUL, right upper lobe.

Table S3. Comparison of the performance of different deep learning models across three cohorts

| Model | AUC (95% CI) | Accuracy | Sensitivity | Specificity | PPV | NPV |
| --- | --- | --- | --- | --- | --- | --- |
| Training Cohort | | | | | | |
| 2.5D resnet18 | 0.916 (0.886–0.946) | 0.856 | 0.865 | 0.849 | 0.827 | 0.882 |
| 2.5D resnet50 | 0.931 (0.905–0.958) | 0.876 | 0.852 | 0.897 | 0.874 | 0.878 |
| 2.5D resnet101 | 0.919 (0.892–0.946) | 0.838 | 0.877 | 0.805 | 0.791 | 0.887 |
| 2D resnet18 | 0.906 (0.874–0.939) | 0.850 | 0.865 | 0.838 | 0.817 | 0.881 |
| 2D resnet50 | 0.910 (0.877–0.943) | 0.865 | 0.839 | 0.886 | 0.861 | 0.868 |
| 2D resnet101 | 0.924 (0.897–0.950) | 0.844 | 0.884 | 0.811 | 0.797 | 0.893 |
| 3D resnet18 | 0.868 (0.827–0.908) | 0.826 | 0.858 | 0.800 | 0.782 | 0.871 |
| 3D resnet50 | 0.839 (0.792–0.885) | 0.824 | 0.806 | 0.838 | 0.806 | 0.838 |
| 3D resnet101 | 0.872 (0.833–0.911) | 0.815 | 0.865 | 0.773 | 0.761 | 0.872 |
| Internal Validation Cohort | | | | | | |
| 2.5D resnet18 | 0.869 (0.809–0.928) | 0.816 | 0.739 | 0.885 | 0.850 | 0.793 |
| 2.5D resnet50 | 0.874 (0.815–0.933) | 0.844 | 0.841 | 0.846 | 0.829 | 0.857 |
| 2.5D resnet101 | 0.852 (0.786–0.919) | 0.816 | 0.826 | 0.808 | 0.792 | 0.840 |
| 2D resnet18 | 0.817 (0.744–0.890) | 0.789 | 0.710 | 0.859 | 0.817 | 0.770 |
| 2D resnet50 | 0.840 (0.771–0.908) | 0.823 | 0.826 | 0.821 | 0.803 | 0.842 |
| 2D resnet101 | 0.814 (0.737–0.890) | 0.796 | 0.797 | 0.795 | 0.775 | 0.816 |
| 3D resnet18 | 0.794 (0.716–0.872) | 0.776 | 0.696 | 0.846 | 0.800 | 0.759 |
| 3D resnet50 | 0.762 (0.678–0.845) | 0.776 | 0.783 | 0.769 | 0.750 | 0.800 |
| 3D resnet101 | 0.753 (0.667–0.839) | 0.762 | 0.768 | 0.756 | 0.736 | 0.787 |
| External Test Cohort | | | | | | |
| 2.5D resnet18 | 0.838 (0.757–0.920) | 0.843 | 0.811 | 0.864 | 0.796 | 0.875 |
| 2.5D resnet50 | 0.834 (0.759–0.909) | 0.821 | 0.774 | 0.852 | 0.774 | 0.852 |
| 2.5D resnet101 | 0.826 (0.748–0.905) | 0.784 | 0.811 | 0.765 | 0.694 | 0.861 |
| 2D resnet18 | 0.818 (0.733–0.902) | 0.828 | 0.811 | 0.840 | 0.768 | 0.872 |
| 2D resnet50 | 0.801 (0.721–0.882) | 0.799 | 0.774 | 0.815 | 0.732 | 0.846 |
| 2D resnet101 | 0.815 (0.735–0.895) | 0.776 | 0.811 | 0.753 | 0.683 | 0.859 |
| 3D resnet18 | 0.778 (0.686–0.869) | 0.806 | 0.792 | 0.815 | 0.737 | 0.857 |
| 3D resnet50 | 0.755 (0.667–0.843) | 0.769 | 0.755 | 0.778 | 0.690 | 0.829 |
| 3D resnet101 | 0.764 (0.675–0.853) | 0.746 | 0.792 | 0.716 | 0.646 | 0.841 |

CI, confidence interval; NPV, negative predictive value; PPV, positive predictive value.

## Supplementary Figures





Figure S1. Heat maps of local features and the derived habitat subregions for a representative case. The first 19 panels respectively display heat maps of individual local features. The last panel shows the habitat subregions generated by clustering the 19 features within the tumor.


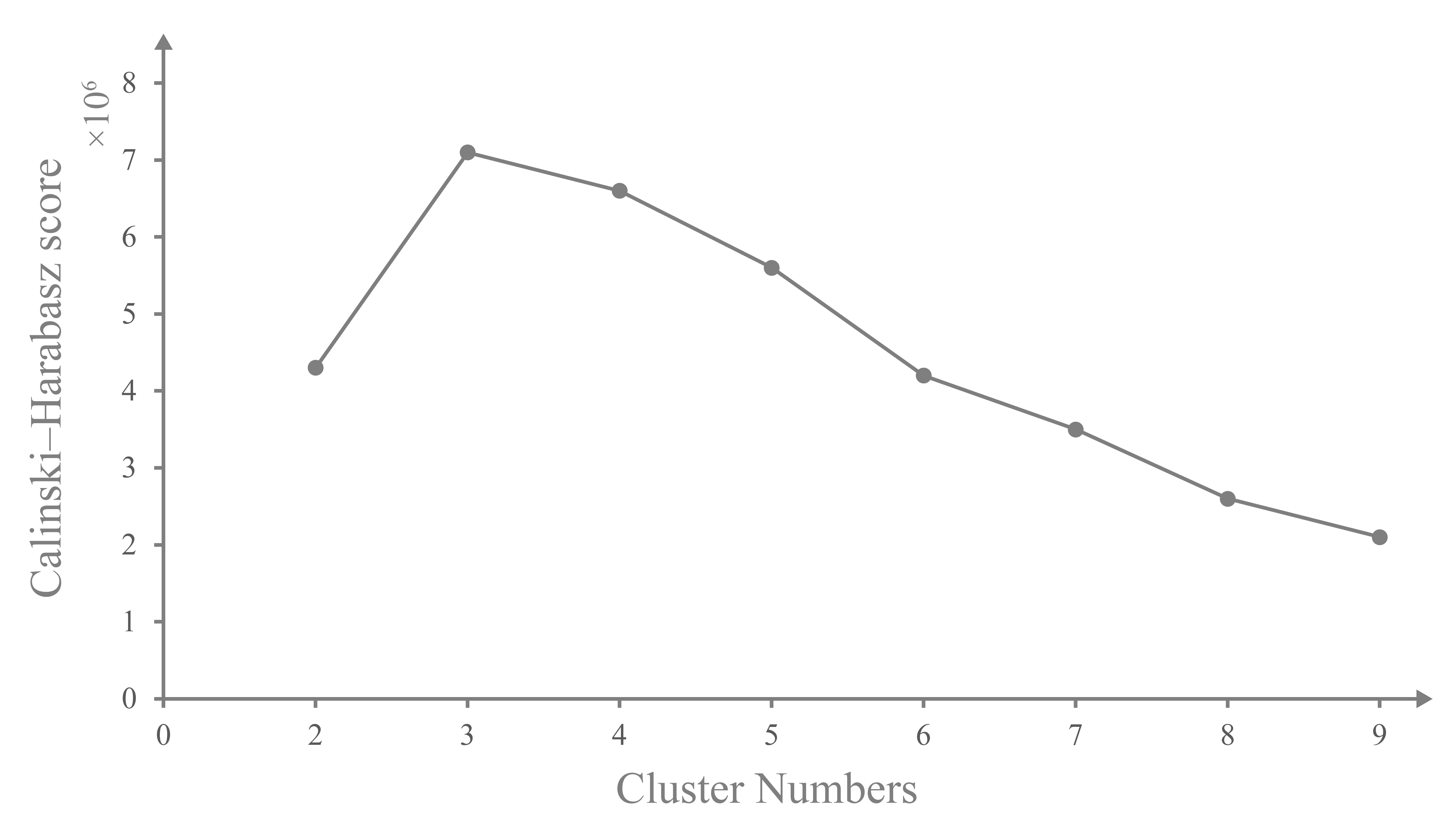


Figure S2. Line plot of the Calinski–Harabasz score as a function of the number of clusters.


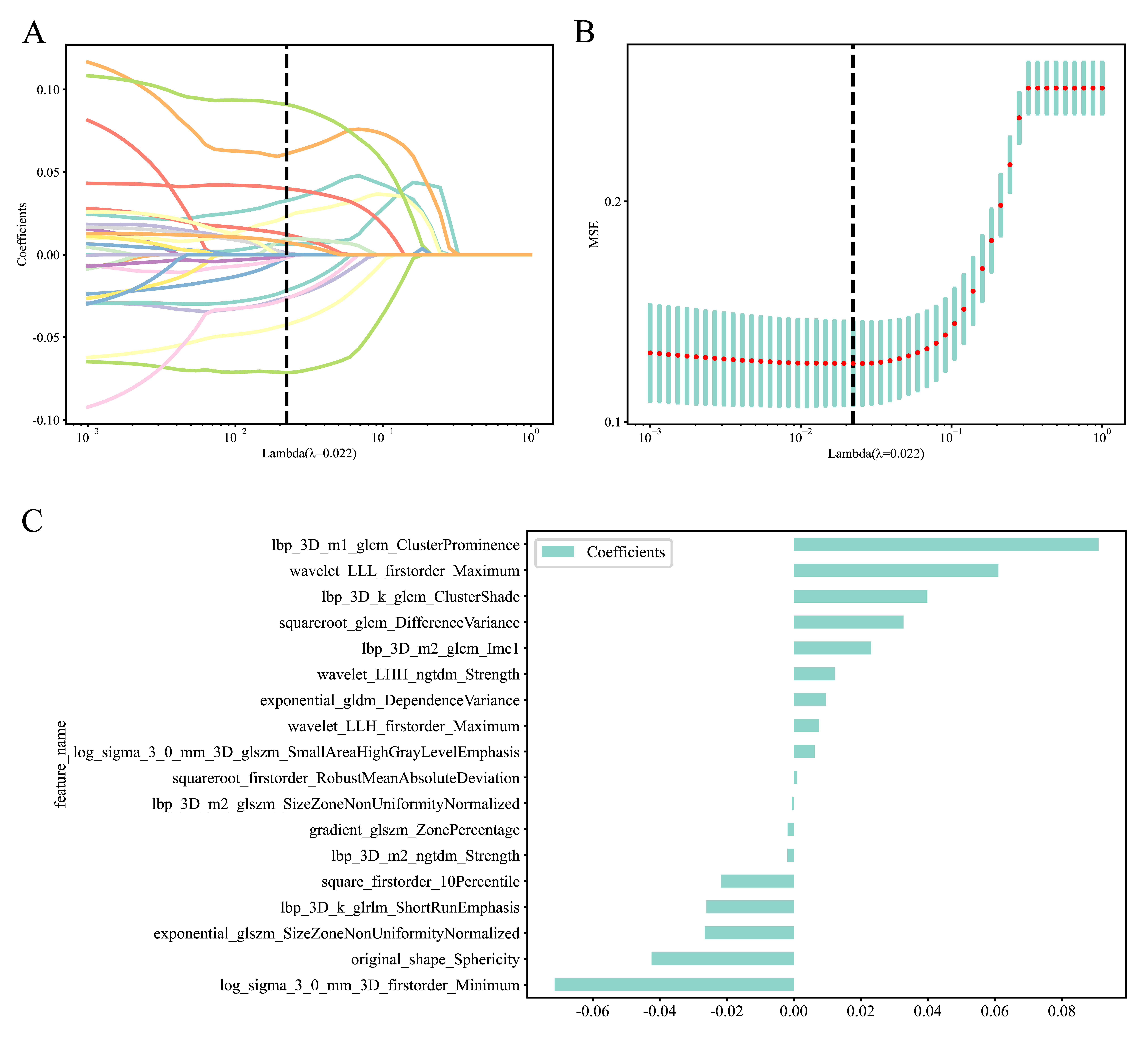


Figure S3. Least absolute shrinkage and selection operator (LASSO)-based radiomics features selection. (A) LASSO coefficients path plot. As the regularization parameter λ increases, the regression coefficients of each radiomics feature are gradually compressed to zero. The vertical dashed line indicates the optimal λ (0.022) selected by 10-fold cross-validation. (B) 10-fold cross-validation MSE curve. Green error bars represent standard deviations, and the red dot denotes the average MSE. The dashed line corresponds to the minimum MSE (λ = 0.022). (C) Bar plot of non-zero feature coefficients at the optimal λ. This plot lists the features with non-zero regression coefficients at the optimal λ, which will be used for subsequent radiomics model construction. MSE, mean squared error.


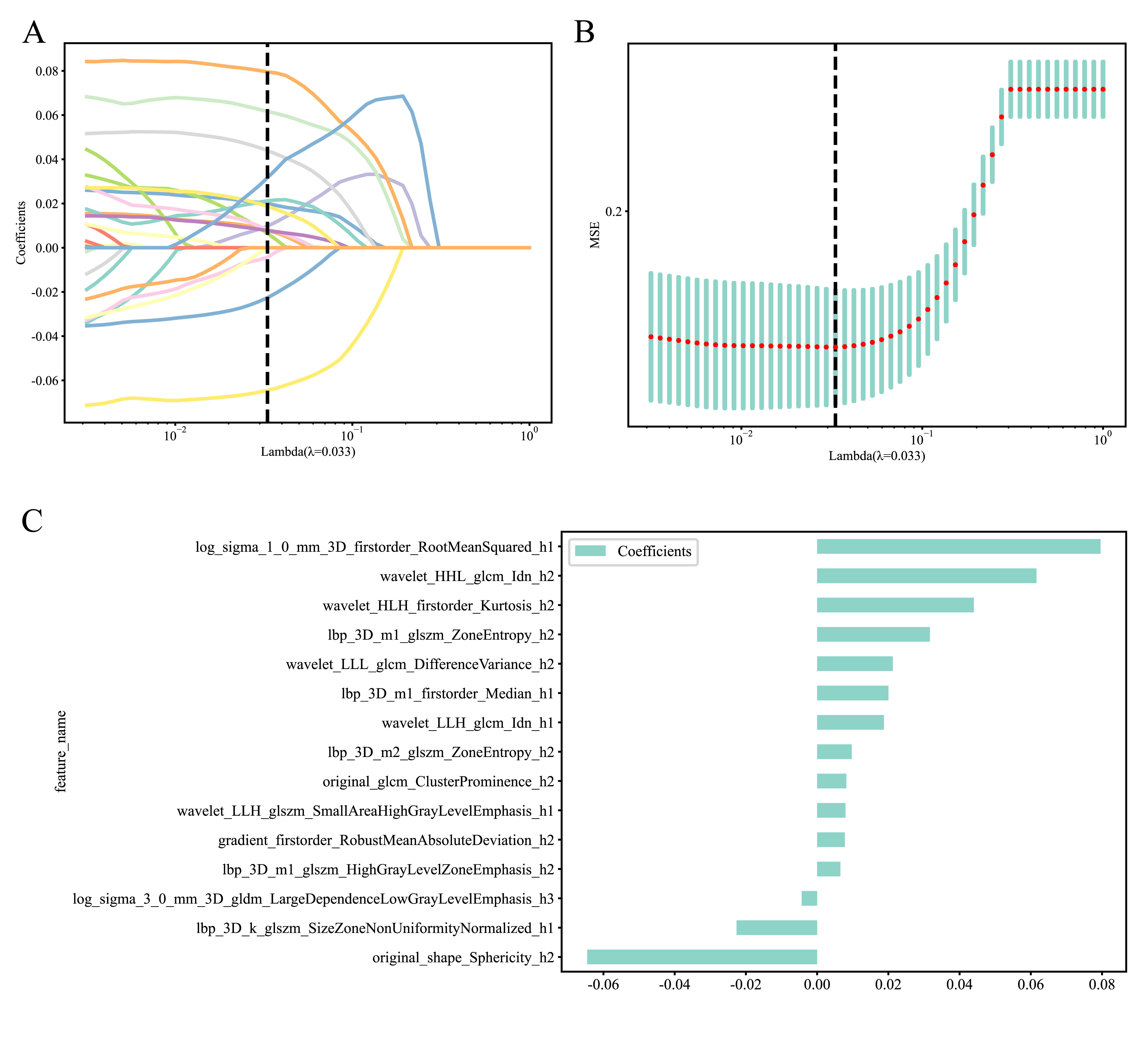


Figure S4. Least absolute shrinkage and selection operator (LASSO)-based habitat features selection. (A) LASSO coefficients path plot. As the regularization parameter λ increases, the regression coefficients of each habitat feature are gradually compressed to zero. The vertical dashed line indicates the optimal λ (0.033) selected by 10-fold cross-validation. (B) 10-fold cross-validation MSE curve. Green error bars represent standard deviations, and the red dot denotes the average MSE. The dashed line corresponds to the minimum MSE (λ = 0.033). (C) Bar plot of non-zero feature coefficients at the optimal λ. This plot lists the features with non-zero regression coefficients at the optimal λ, which will be used for subsequent habitat model construction. MSE, mean squared error.


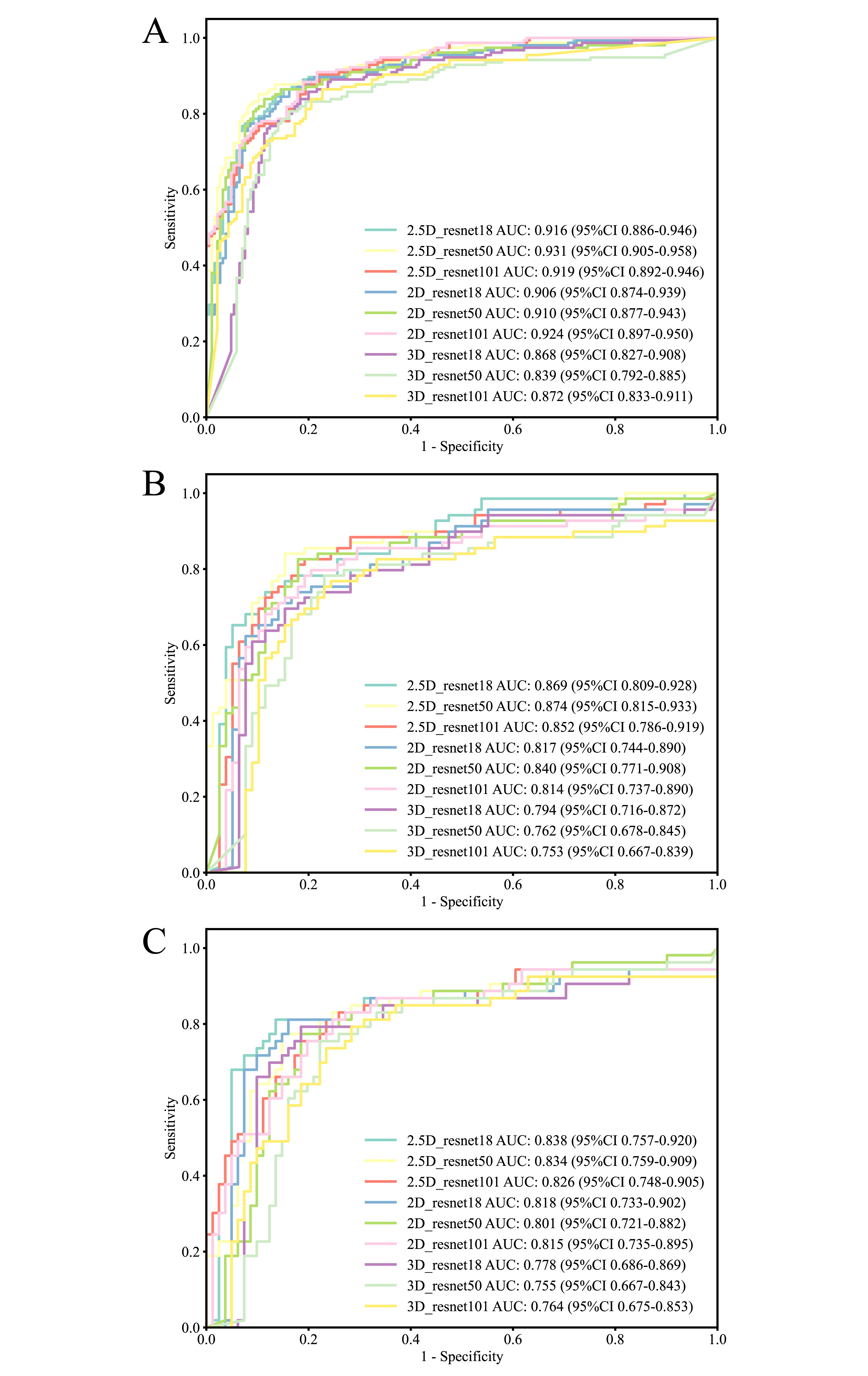


Figure S5. Receiver operating characteristic (ROC) curves of the deep learning models across the three cohorts. (A) ROC curves of the deep learning models in the training cohort. (B) ROC curves of the deep learning models in the internal validation cohort. (C) ROC curves of the deep learning models in the external test cohort.


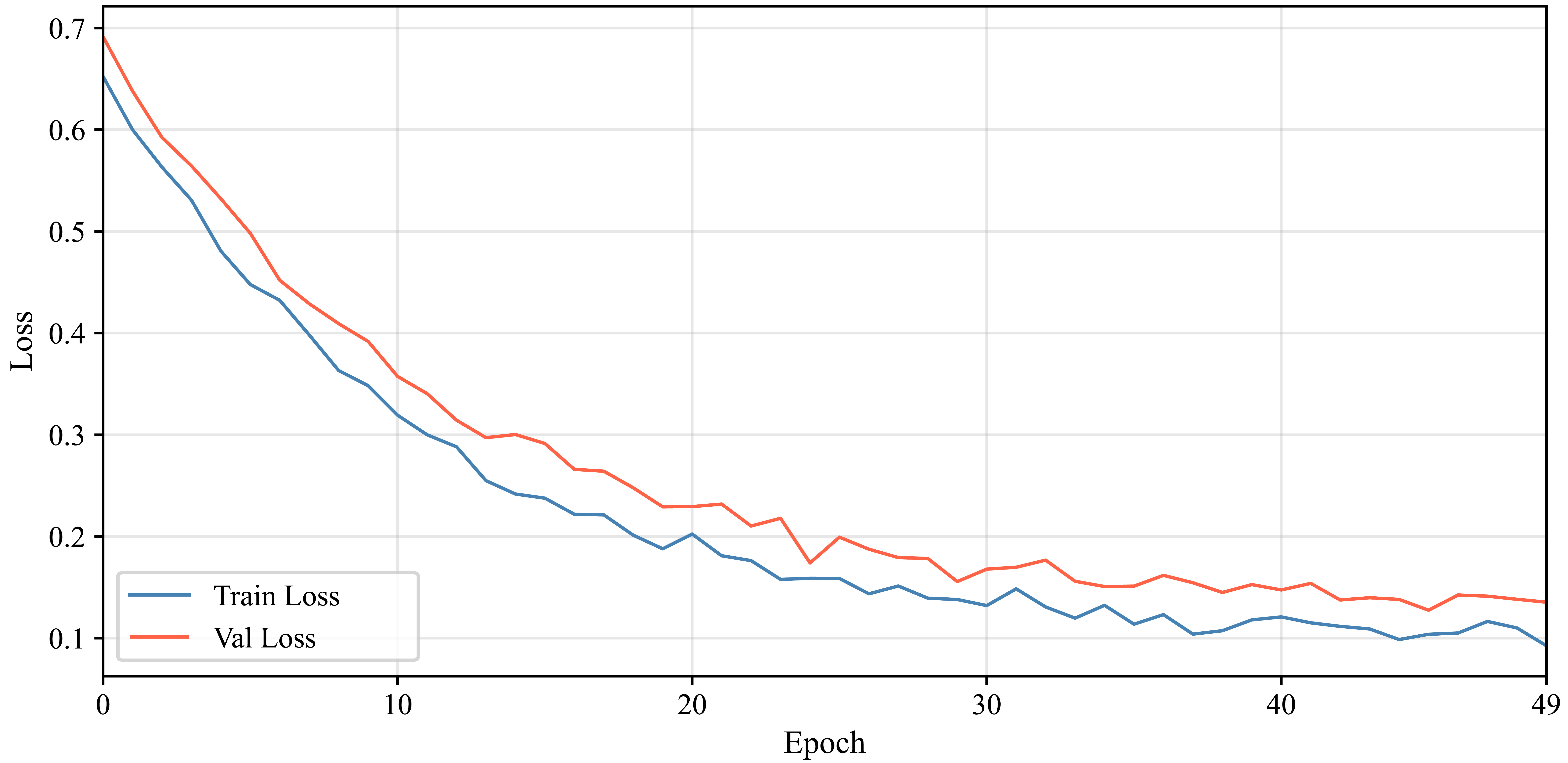


Figure S6. Training and validation loss curves of the optimal deep learning model (2.5D ResNet50)


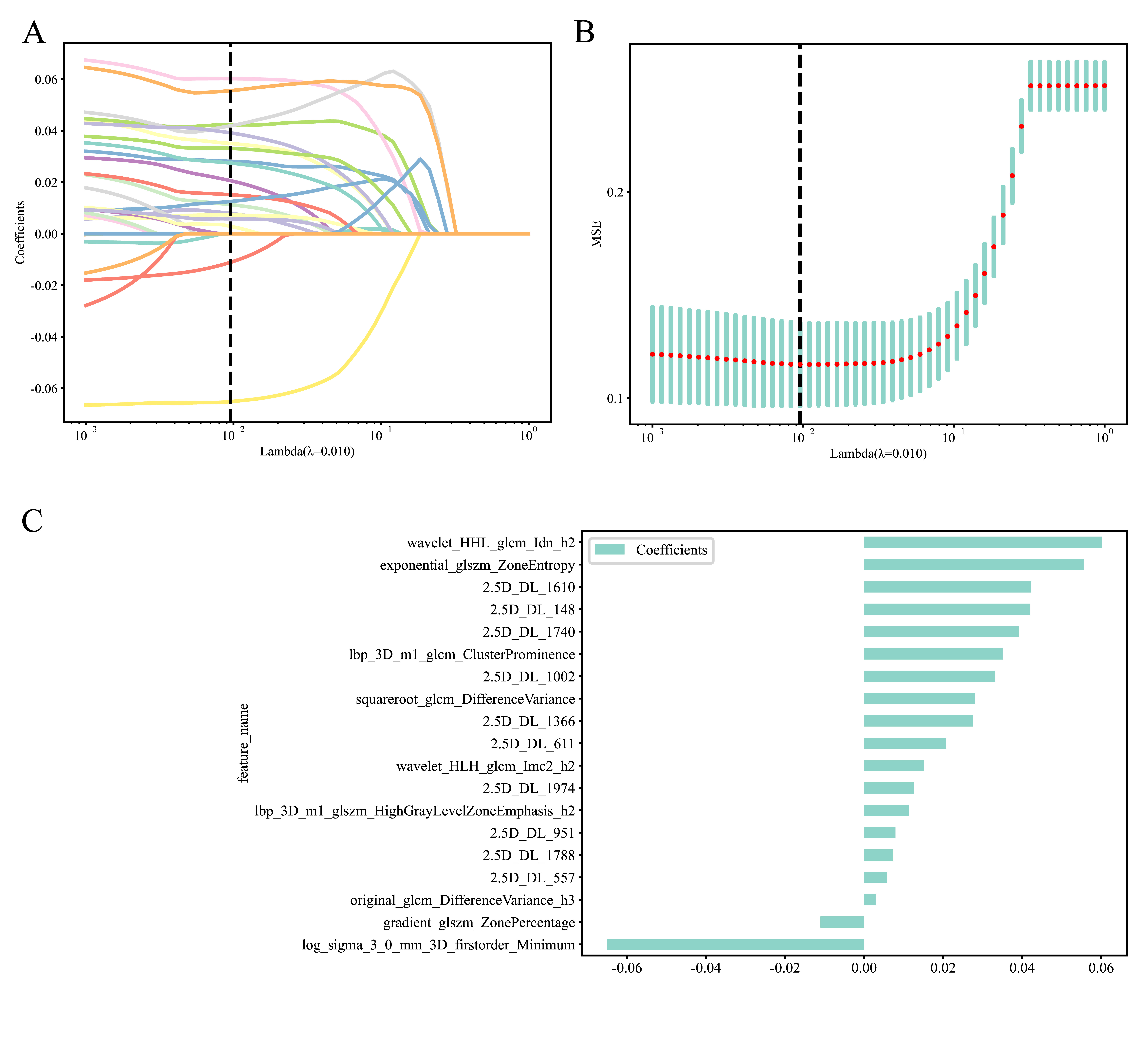


Figure S7. Least absolute shrinkage and selection operator (LASSO)-based fusion features selection. (A) LASSO coefficients path plot. As the regularization parameter λ increases, the regression coefficients of each fusion feature are gradually compressed to zero. The vertical dashed line indicates the optimal λ (0.010) selected by 10-fold cross-validation. (B) 10-fold cross-validation MSE curve. Green error bars represent standard deviations, and the red dot denotes the average MSE. The dashed line corresponds to the minimum MSE (λ = 0.010). (C) Bar plot of non-zero feature coefficients at the optimal λ. This plot lists the features with non-zero regression coefficients at the optimal λ, which will be used for subsequent fusion model construction. MSE, mean squared error.


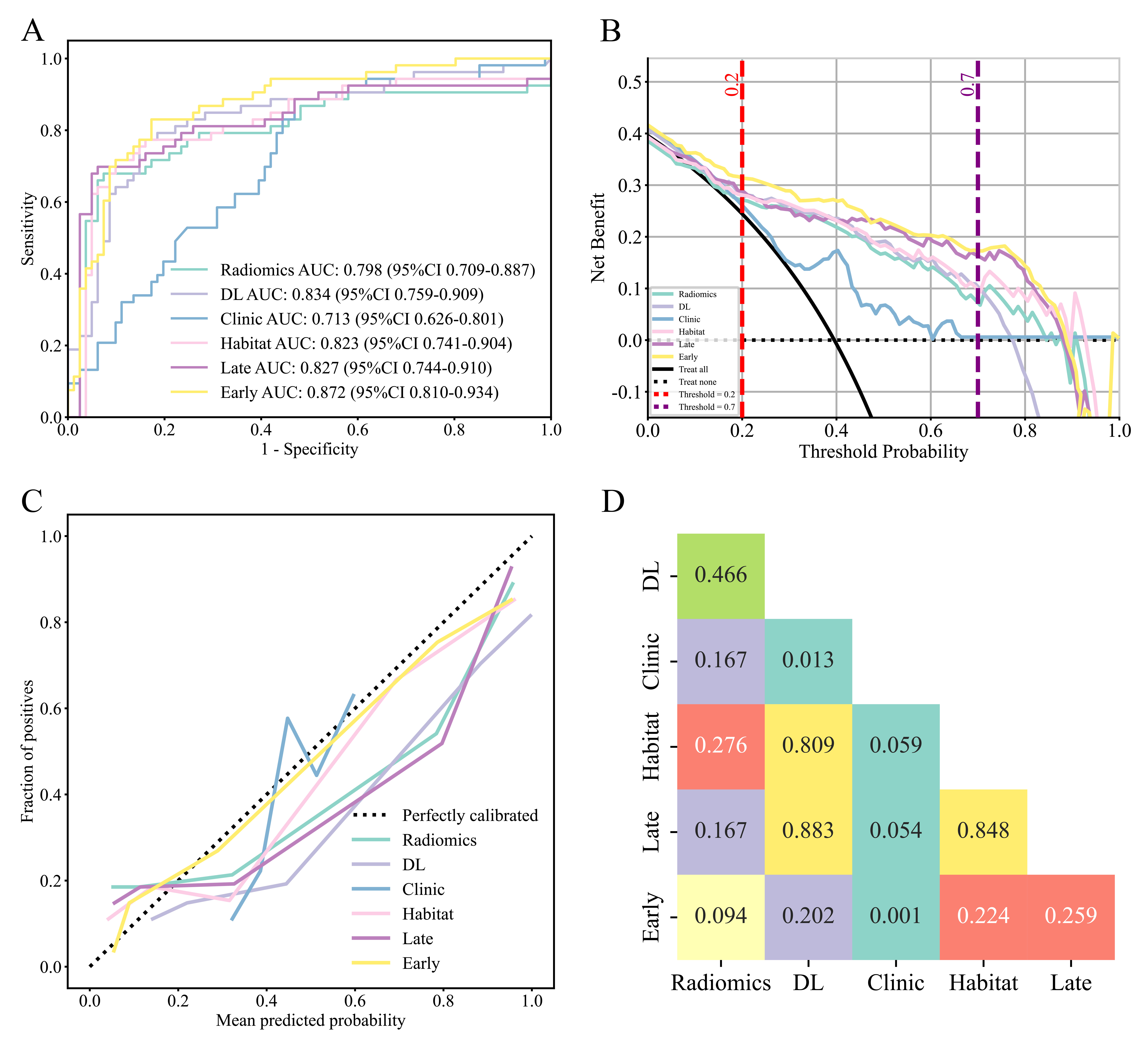


Figure S8. Performance evaluation and comparison of different models in the external test cohort. (A) Receiver operating characteristic curves of different models. (B) Decision curve analysis of different models. (C) Calibration curves of different models. (D) DeLong test among different models. DL, deep learning.
